# Pirin Inhibits FAS‐Mediated Apoptosis to Support Colorectal Cancer Survival

**DOI:** 10.1002/advs.202301476

**Published:** 2023-12-26

**Authors:** Huanhuan Ma, Muhammad Suleman, Fengqiong Zhang, Tingyan Cao, Shixiong Wen, Dachao Sun, Lili Chen, Bin Jiang, Yue Wang, Furong Lin, Jinyang Wang, Boan Li, Qinxi Li

**Affiliations:** ^1^ State Key Laboratory of Cellular Stress Biology, School of Life Sciences, Faculty of Medicine and Life Sciences Xiamen University Xiamen 361102 China

**Keywords:** Apoptosis, colon cancer, FAS, NFκB2, pirin

## Abstract

Resistance to immunotherapy in colorectal cancer (CRC) is associated with obstruction of FAS (Apo‐1 or CD95)‐dependent apoptosis, a hallmark of cancer. Here it is demonstrated that the upregulation of pirin (PIR) protein in colon cancers promotes tumorigenesis. Knockout or inhibition of PIR dramatically increases FAS expression, FAS‐dependent apoptosis and attenuates colorectal tumor formation in mice. Specifically, NFκB2 is a direct transcriptional activator of FAS and robustly suppressed by PIR in dual mechanisms. One is the disruption of NFκB2 complex (p52‐RELB) association with *FAS* promoter, the other is the inhibition of NIK‐mediated NFκB2 activation and nuclear translocation, leading to the inability of active NFκB2 complex toward the transcription of FAS. Furthermore, PIR interacts with FAS and recruits it in cytosol, preventing its membrane translocation and assembling. Importantly, knockdown or knockout of PIR dramatically sensitizes cells to FAS mAb‐ or active CD8^+^ T cells‐triggered cell death. Taken together, a PIR‐NIK‐NFκB2‐FAS survival pathway is established, which plays a key role in supporting CRC survival.

## Graphical abstract

Upregulated pirin (PIR) facilitates colorectal cancer (CRC) survival by suppressing Fas‐mediated apoptosis in two ways, namely, the disruption of NFκB2‐driven FAS transcription and the attenuation of FAS membrane translocation and assembling. Downregulation or inhibition of PIR dramatically sensitizes CRC cells to killing activity of CD8^+^ T cell and promotes FAS‐dependent apoptosis, suggesting the potential of PIR as an anti‐CRC target.

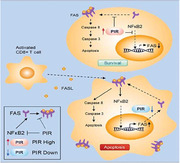

## 1 Introduction

PIR (Pirin), a member of the cupin superfamily protein, was initially identified as an interactor of nuclear factor I/CCAAT box transcription factor (NFI/CTF1) in a yeast two‐hybrid screen.^[^ ^1^ ^]^ It is universally expressed in almost all tissues of human body and predominantly localized in nucleus and cytoplasm. In recent years, emerging evidence has linked PIR to several types of cancers including colon cancer,^[^ ^2^ ^]^ breast cancer^[^ ^3^ ^]^ and melanoma,^[^ ^4^ ^]^ where it may act as a stimulator to cancer growth and malignancy.^[^ ^4^, ^5^ ^]^ One line of evidence supporting this role of PIR is the observation that PIR functions as a transcriptional coregulator of nuclear factor kappa‐light‐chain‐enhancer of activated B cells 1 (NFκB1) to facilitate its DNA binding ability and transcriptional activity in vitro.^[^ ^6^ ^]^ Moreover, PIR has been found to act as an iron‐dependent redox regulator of RELA (p65), a component of NFκB1 complex, thereby enhancing its DNA‐binding ability.^[^ ^7^ ^]^ In addition, PIR has been identified as a target of NRF2,^[^ ^2^, ^8^ ^]^ and is overexpressed in human colorectal cancer with unknown functions.^[^ ^2^ ^]^ Colorectal cancer is a kind of malignant tumors that originates from the colon or rectum. In this study, we aimed to identify the precise biological functions and corresponding mechanisms of PIR in promoting tumorigenesis in CRC. To address these questions, we knocked down (KD) PIR in HCT116 cells and observed severe apoptosis and dramatic FAS upregulation, demonstrating that PIR may be a suppressor of FAS expression.

FAS is a prototypical apoptosis‐inducing death receptor in the tumor necrosis factor receptor (TNFR) superfamily.^[^ ^9^ ^]^ Once binding to FAS ligand (FASL) or accumulating on membrane, FAS trimerizes automatically and sequentially activates various intermediary proteins including FADD, caspase8 and caspase3, leading to apoptosis eventually.^[^ ^10^ ^]^ As a result, the FAS‐dependent apoptosis of cancer cells triggered by activated CD8^+^ T cell is considered as an important immunosurveillance of host malignancy. However, cancer cells usually acquire ability to escape FAS‐medicated apoptosis by downregulating FAS expression^[^ ^11^ ^]^ or inhibiting its cell surface localization.^[^ ^12^ ^]^ In this regard, PIR suppression of FAS expression may confer cancer cells ability to escape from FAS‐dependent immunosurveillance. Considering PIR is a negative regulator of FAS expression and a coregulator of NFκB1, we then focused on whether PIR inhibits FAS expression via regulating NFκB pathway.

The NFκB family consists of transcriptional factors RELA (p65), RELB, c‐REL, NFκB1 (p105/p50) and NFκB2 (p100/p52), which belong to two major pathways: the canonical and the non‐canonical NFκB pathways.^[^ ^9^, ^13^ ^]^ Activation of the canonical NFκB1 pathway depends on the proteasomal degradation of IκBα, leading to the activation of the protein complex composed of p50 and p65/c‐REL.^[^ ^14^ ^]^ On the contrary, activation of the non‐canonical or alternative NFκB2 pathway largely relies on the selectively proteasomal degradation of p100 precursor (termed p100 processing) to yield the protein fragment p52 which forms heterodimer with RELB. p52/RELB complex then acts as a functional transcription factor.^[^ ^9^, ^15^ ^]^ NFκB‐inducing kinase (NIK) plays a key role in p100 processing and activation. NIK tightly integrates signals from a series of TNF receptor family members and activates the downstream kinase, IκB kinase α (IKKα), which sequentially triggers p100 phosphorylation and processing, resulting in non‐canonical NFκB2 activation. NFκB2 was reported to function as a pro‐apoptotic protein upon certain death‐inducing signals’ stimulation,^[^ ^15^, ^16^ ^]^ in spite of its main functions in regulating immune response.^[^ ^14c^ ^]^

Following above clues, we investigated in depth the molecular mechanism underlying PIR inhibition of cancer cell apoptosis and found that NFκB2 rather than NFκB1 is involved in PIR regulation of apoptosis by establishing the PIR‐NIK‐NFκB2‐FAS regulatory axis. Through this axis, overexpressed PIR inhibits NFκB2 transcriptional activity toward FAS and therefore assists cancer cells surviving from FAS‐dependent insults, especially from immune defense system. Moreover, PIR interacts with FAS in cytosol and prevents FAS from translocating to membrane. Upon inhibition or knockdown of PIR, FAS accumulates in membrane and is activated automatically. Active FAS then stabilizes NIK and consequently triggers the transcriptional activity of NFκB2 toward FAS, establishing a feedforward loop for amplification of FAS death signaling. Our research therefore reinforces the importance of targeting PIR as a therapeutic strategy to overcome the resistance of CRC, particularly those with PIR‐mediated FAS downregulation, to immunotherapy.

## 2 Results

### 2.1 PIR Deficiency Triggers Apoptosis

**Figure 1:**
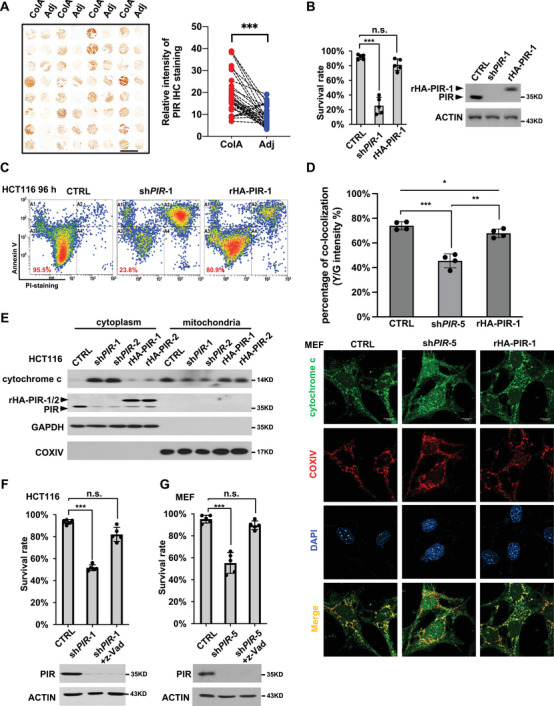
PIR deficiency triggers apoptosis. A) IHC staining of successive colon tissue microarrays with PIR antibody (left) and the PIR protein expression intensity analysis (right). ColA: Hcol‐Ade carcinoma; Adj: tumor‐adjacent normal colon tissue. Scale bars represent 3 mm. Data are analyzed employing paired Student's *t*‐test (***p < 0.001, n = 32). B and C) HCT116 cell were infected with lentiviruses expressing sh*PIR*‐1 to knock down endogenous PIR and further rescued for PIR expression with rescuing expression of HA‐tagged PIR‐1 (rHA‐PIR‐1) whose sh*PIR*‐1 targeting sequence contain synonymous mutations and resistant to sh*PIR*‐1. After 96 hours of infection, survival rates were determined employing Annexin V‐fluorescein isothiocyanate (FITC)/propidium iodide (PI) staining‐based flow cytometry C). PIR protein was detected by Western blot (WB) (B right). Data are presented as mean±SD of five independent experiments and analyzed employing unpaired Student's *t*‐test (B left, ***p < 0.001, n.s.: no significant difference).  CTRL: control. D) MEF cells were treated as in B, followed by immunostaining to show cytochrome c and COXIV localization (lower panel). The statistics (upper panel) are presented as mean±SD of four independent experiments (unpaired Student's *t*‐test, *p < 0.05, **p < 0.01, ***p < 0.001). Nuclei were stained using DAPI. Scale bars represent 10 µm. Y/G refers to Yellow/Green. E) HCT116 cells with PIR KD or rescuing expression were isolated for cytoplasm and mitochondria fractions, followed by detection of cytochrome c, GAPDH (as a cytosol control) and COXIV (as a mitochondria control). F and G) HCT116 cells (F) and MEF cells (G) were infected with lentiviruses expressing sh*PIR‐1* or sh*PIR‐5*, followed by treatment with pan‐caspase inhibitor Z‐VAD‐FMK. Seventy two hours post‐infection, cells were stained with PI and counted with flow cytometer for survival rate (upper panel). PIR KD efficiency was determined by WB (lower panel). Results are presented as mean±SD of five independent experiments (unpaired Student's *t*‐test, ***p < 0.001, n.s.: no significant difference).

Our tumor tissue microarray IHC data (**Figure** **1**A) and the open access clinical dataset (Figure S1A, Supporting Information) indicate that PIR is upregulated in CRC at both protein and mRNA levels. Moreover, PIR expression level is negatively correlated with survival rate in CRC (Figure S1B, Supporting Information), implying that PIR may play a role in stimulating the malignancy of CRC. To clarify the mechanism underlying such role of PIR, we knocked down PIR with short hairpin RNA against *PIR* (sh*PIR*) in colorectal cells HCT116, CT26 and HT29 and mouse embryonic fibroblast (MEF), further rescued its expression with HA‐tagged rescuing plasmids (rHA‐PIR), and evaluated the survival state of these cells. Surprisingly, PIR knockdown (KD) caused clear death morphology and significant apoptosis as determined by Annexin V‐PI staining, and such apoptosis could be rescued by re‐expression of PIR (Figure 1B, C; Figure S1C, Supporting Information), indicating that PIR may serve to maintain cell survival by suppressing apoptosis. To confirm this observation, we detected cytochrome c release and mitochondrial polarization. Knockdown of PIR promoted the release of cytochrome c (Figure 1D,E) and mitochondrial depolarization which is indicated by an increase in the ratio of green fluorescence intensity of JC‐1 dye (Figure S1D, Supporting Information), while such alterations were reversed by rescuing expression of PIR. Moreover, pan‐caspase inhibitor z‐VAD‐FMK completely blocked cell death induced by PIR knockdown in both HCT116 cells (Figure 1F) and MEFs (Figure 1G). These results prove that loss of PIR triggers mitochondrial cell apoptotic pathway, revealing a pivotal role of PIR in maintaining cell survival, a function different from its role in cell metastasis identified previously.^[^ ^17^ ^]^

### 2.2 PIR Deficiency‐Triggered Apoptosis is Mediated by Activation of FAS Transcriptional Activities

**Figure 2:**
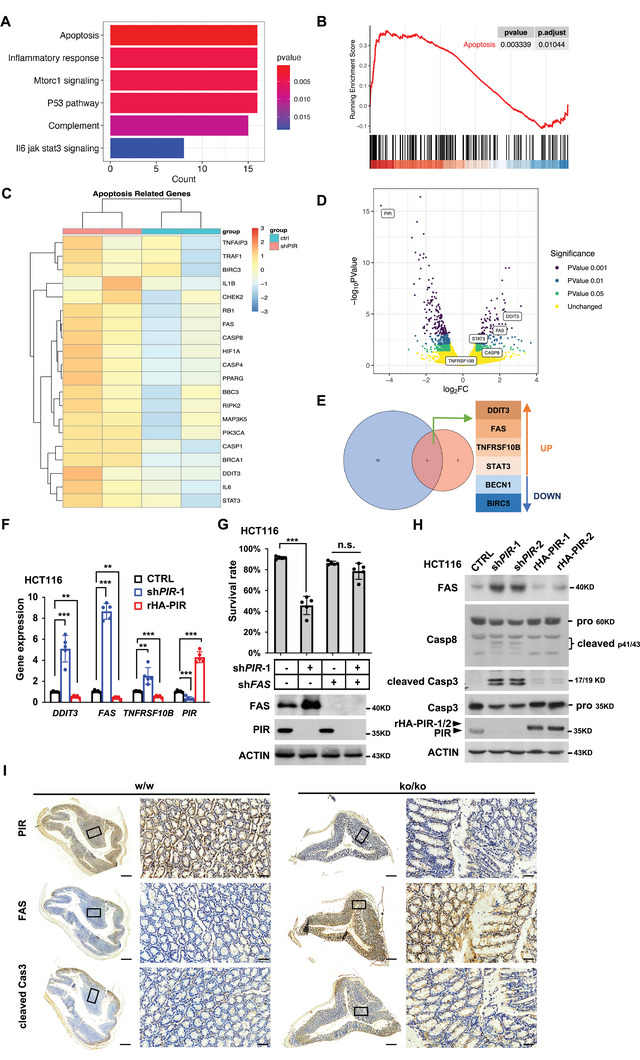
PIR deficiency‐triggered apoptosis is mediated by upregulation of FAS transcriptional activities. A) Bar charts for MSigDB's hallmark collection enrichment analysis of differentiated genes in RNA‐Seq data of PIR KD HCT116 cells in this study (|log2FC| ≥ 1, *p*‐value < 0.05). B) GSEA enrichment of the RNA‐Seq data of HCT116 cells in this study for signatures of apoptosis pathway. *P* values were determined by one‐tailed permutation test by GSEA. C) Expression heatmap of the most upregulated apoptotic genes in RNA‐Seq data generated in this study. D) Volcano plot of the transcriptome DEGs in PIR knockdown group compared to control group. The abscissa represents the log_2_FoldChange (log_2_FC) of gene expression between different groups. The ordinate represents the significance level of the expression difference. DEGs were calculated using the limma voom R package. E) Venn diagram showing most changed apoptotic genes sharing between our PIR KD RNA‐Seq data and publicly available PIR KD expression dataset (GSE17551 and GSE16798). F) HCT116 cells were knocked down and further rescued for PIR expression. After 48 hours of PIR KD, total RNA was extracted, followed by determination of indicated genes’ expression using RT‐qPCR. Data are shown as mean±SEM (n = 5, unpaired Student's *t*‐test, *p < 0.05, **p < 0.01, ***p < 0.001). G) HCT116 cells were knocked down for *PIR*‐1 and *FAS* alone or in combination. After 72 hours of knockdown, cells were stained with PI and then subjected to survival analysis by flow cytometry. Data represent mean±SD of five independent experiments (unpaired Student's *t*‐test, ***p < 0.001, n.s.: no significant difference). H) WB was performed to determine the protein levels of FAS, PIR, full length of casp8 and casp3, and cleaved fragments of Casp8 and Casp3 in HCT116 cells with PIR KD or rescuing expression. Casp refers to caspase. I) Representative images of PIR, FAS and cleaved Cas3 IHC staining in the colon of PIR KO mice. Scale bars, 500 µm for low magnification (1 ×, left panel), and 50 µm for high magnification (10 ×, right panel).

To determine the mechanism of PIR KD‐induced cell death, we performed the RNA‐Seq of HCT116 cells after PIR KD. Further enrichment analysis of RNA‐Seq result using Gene Set Enrichment Analysis (GSEA)^[^ ^18^ ^]^ identified significant enrichment of apoptosis related gene set in PIR KD cells (**Figure** **2**A,B). Corresponding heat map (Figure 2C) as well as volcano plot (Figure 2D) showed that genes involved in death receptor‐mediated apoptotic pathway, such as *FAS*, *TNFRSF10B*, and *CASPs* were among the most upregulated apoptotic genes. Consistently, these apoptosis related genes were also markedly upregulated in *PIR* KD U937 (GSE17551) and WM266 (GSE16798)^[^ ^5b^ ^]^ cells as indicated by analysis of the transcriptome profile using the data from the Gene Expression Omnibus (GEO) databases (Figure S2A,B, Supporting Information). Next, we compared top upregulated and downregulated genes from our RNA‐Seq data and above data sets for the most significantly changed apoptotic genes and found that *DDIT3, FAS* and *TNFRSF10B* were the overlapping genes (Figure 2E). This observation was further confirmed by qRT‐PCR in HCT116 cells (Figure 2F) and MEFs (Figure S2C, Supporting Information). It is worth noting that the MEFs used here were Large T transformed and characterized by immortalization, enhanced proliferation, loss of contact inhibition and altered gene expression profile. Such MEFs therefore provide a model system that allows us to investigate fundamental aspects of cell biology and explore the molecular mechanisms underlying various disease including cancer. Considering *DDIT3* and *FAS* were upregulated far higher than *TNFRSF10B* in these two *PIR* KD cell lines and the pro‐apoptotic function of them had been well documented,^[^ ^19^ ^]^ we therefore checked which of them play the key role in PIR deficiency‐triggered apoptosis. Interestingly, further knockdown of *FAS* rather than *DDIT3*, completely blocked PIR depletion‐induced cell death in both HCT116 (Figure 2G; Figure S2F, Supporting Information) and MEF cells (Figure S2D,E, Supporting Information), indicating that FAS may be the main downstream apoptotic factor of PIR. Moreover, we examined 54 paired publicly available human CRC patient‐derived data from GEO database and found that FAS expression level is much lower in colon cancer tissue than in corresponding adjacent colon tissue and negatively correlated with PIR expression level, implying that FAS expression may be suppressed by PIR (Figure S2G, Supporting Information). In contrast, DDIT3 expression is not evidently altered in CRC and show no close correlation with PIR expression (Figure S2H, Supporting Information). These results suggest that upregulated FAS expression is required for PIR deficiency‐triggered cell death. It is well known that FAS plays a key role in extrinsic apoptosis pathway.^[^ ^11a^ ^]^ Upon activation, FAS undergoes trimerization and forms the death‐inducing signaling complex (DISC) with FADD and caspase 8, where pro‐caspase 8 is cleaved to produce active caspase 8 which in turn activates caspase 3, and eventually leads to apoptosis.^[^ ^10d^ ^]^ We examined cleavage status of caspase 8 and caspase 3 in PIR KD cells. As expected, considerable cleavages of caspase 8 and caspase 3 were observed in PIR KD HCT116 (Figure 2H), MEF and HT29 cells (Figure S2I, Supporting Information), in consistence with FAS upregulation, and this phenomenon was completely reversed by rescuing expression of PIR (Figure 2H). It is worth mentioning that we crossed conditional PIR KO mice with *Villin Cre‐ERT2*, in which Cre‐mediated gene deletion occurs in intestinal epithelial cells after tamoxifen administration. Mice were sacrificed one‐month post‐induction. The colon of PIR KO mice showed significantly upregulation of FAS and cleaved caspase 3 expression (Figure 2I). Taken together, increased PIR expression may contribute to survival of CRC by suppressing FAS death pathway.

### 2.3 PIR Suppresses FAS Transcriptional Activities by Disrupting The Binding of NFκB2 to FAS Promoter

Since *FAS* mRNA level was downregulated by PIR, we next sought to clarify the potential mechanism underlying such regulation. By creating a luciferase reporter construct containing full length human *FAS* promoter (‐1235 bp to +1 bp) (Figure S3A, Supporting Information), we found that PIR protein efficiently inhibited luciferase activity of this reporter in a dose dependent manner (Figure S3B, Supporting Information). On the contrary, treatment of cells with a reported PIR inhibitor CCG‐1423 enhanced *FAS* luciferase activity dramatically (Figure S3C, Supporting Information). These results indicated that PIR suppresses *FAS* transcriptional activities. To determine the exact region on *FAS* promoter responsible for PIR inhibition, we constructed a series of luciferase reporters based on *FAS* promoter deletions (Figure S3A, Supporting Information). Unexpectedly, among all mutants, *FAS*‐Δ3′ (‐604 bp to +1 bp) (Figure S3D, Supporting Information), *FAS*‐Δ3′‐2 (deletion of 101 bp from ‐501 bp to ‐401 bp) (Figure S3E, Supporting Information) and *FAS*‐Δ3′‐2a (deletion of 30 bp from ‐501 bp to ‐472 bp) (Figure S3F, Supporting Information) almost lost luciferase activity and thus never be further suppressed by PIR. These observations raise a possibility that there may exist a putative transcriptional activator for FAS which may directly activate FAS transcriptional activities by binding to its 3′‐2a region (‐501 bp to ‐472 bp) and PIR may not participate directly in such regulation. Indeed, an in vitro EMSA experiment indicates that GST‐PIR protein failed to bind to DNA probe containing 3′‐2a region (Figure S3G, Supporting Information), confirming the hypothesis that PIR may suppress *FAS* transcriptional activities indirectly through altering the activity of a certain transcriptional factor governing *FAS*.

**Figure 3:**
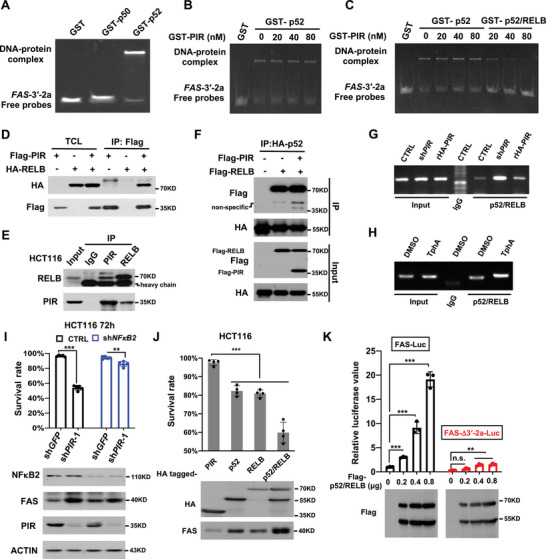
NFκB2 is required for *FAS* transactivation. A) Gel shift assay was performed to observe the retardation of GST, GST‐p50 or GST‐p52 to mobility of DNA probe covering 3′‐2a regions of *FAS* promoter. Probe and protein concentration were 20 nm. B) Gel shift assay was performed with probe 3′‐2a, GST‐p52 protein and increasing doses of GST‐PIR protein. The final concentration of probe was 20 nm. C) Gel shift assay was performed with probe 3′‐2a and indicated proteins. The final concentration of probe was 20 nm. D) Flag‐tagged PIR and HA‐tagged RELB were transiently expressed in HEK293T cells. Co‐IP assay were performed with anti‐Flag agarose (Flag IP), followed by detection of Flag‐PIR and HA‐RELB in the immuno‐precipitates. E) Co‐IP assays were performed with endogenous proteins to determine the association of PIR with RELB in HCT116 cells. F) Flag‐PIR, Flag‐RELB and HA‐p52 were transiently expressed in HEK293T cells. Co‐IP were performed to determine whether PIR disrupts the interaction between RELB and p52. G) Flag‐RELB and Flag‐p52 were transiently expressed in HCT116 cells, followed by knockdown of PIR with sh*PIR* and further reconstitution of PIR with rHA‐PIR. Chromatin immunoprecipitation (ChIP) was then performed to determine the binding of p52/RELB to *FAS* promoter using anti‐Flag antibody. H) HCT116 cells were transfected with Flag‐RELB and Flag‐p52, followed by treatment with DMSO or TphA (50 µm, 12 h). ChIP assays were then performed as in (G). I) HCT116 cells were firstly expressed for sh*GFP* (as control) and sh*NFκB2* individually, to create cell lines with NFκB1 knockdown. 24 hours later, each group of cells were further expressed for sh*GFP* or sh*PIR*‐1. After cultured for another 72 hours, cells were evaluated for survival rate by flow cytometry (upper panel) and expression levels of indicated proteins by WB (lower panel). Data represent the mean±SD. (n = 5, unpaired Student's *t*‐test, **p < 0.01, ***p < 0.001). J) HCT116 cells were transfected with indicated plasmids. After 72 hours of transfection, cells were determined for survival rate (upper panel) and expression levels of proteins (lower panel). Date represents the mean±SD. (n = 4, unpaired Student's *t*‐test, ***p < 0.001). K) HEK293T cells were co‐transfected with luciferase reporter plasmids carrying *FAS* full‐length promoter (*FAS*‐Luc) or its 3′‐2a deletion mutants (*FAS*‐3′‐2a‐Luc), along with different doses of Flag‐p52/RELB complex. After 24 hours of transfection, luciferase activities were determined and normalized to the first column (upper panel). Protein levels were determined by WB (lower panel). Data are presented as mean±SD. (n = 3, unpaired Student's *t*‐test, **p < 0.01, ***p < 0.001, n.s.: no significant difference).

As PIR was previously reported to be a transcriptional cofactor of NFκB1, we determined whether NFκB1 is involved in PIR regulation of FAS expression by performing EMSA assays. Unexpectedly, GST‐tagged p52 (activated form of NFκB2, as a control) rather than p50 (activated form of NFκB1) could bind to 3′‐2a region of *FAS* promoter (**Figure** **3**A), and such binding failed to be further influenced by increasing doses of PIR protein (Figure 3B). As p52 always bind to RELB to conduct their transcriptional activity, we wondered whether PIR influences the association of p52‐RELB complex with *FAS* promoter. Surprisingly, PIR disrupted the binding of p52‐RELB complex towards *FAS* promoter (Figure 3C), implying that PIR may interact with RELB and thus block the interaction of p52‐RELB complex with *FAS*

promoter. Indeed, co‐IP assays showed that PIR interacts with RELB at overexpressed level (Figure 3D) and endogenous level (Figure 3E) and such interaction fails to alter p52‐RELB complex formation (Figure 3F). Moreover, chromatin immunoprecipitation (ChIP) assays indicated that PIR knockdown in HCT116 cells led to an increased binding of p52/RELB complex to *FAS* promoter, and such increase was reversed by reconstitution of PIR expression (Figure 3G). Similarly, treatment of HCT116 cells with PIR inhibitor TphA also enhanced the binding of p52/RELB to *FAS* promoter (Figure 3H). Taking these evidences together, we suggest that PIR interacts with RELB and consequently interferes with the binding of p52‐RELB complex to *FAS* promoter.

To further demonstrate the involvement of NFκB2 in PIR regulation of FAS‐mediated apoptotic pathway, we knocked down NFκB2 expression by shRNA, and found that PIR KD‐triggered cell death as well as FAS upregulation was effectively blocked (Figure 3I), indicating that NFκB2 may play a key role in PIR KD‐triggered cell death by stimulating FAS expression. Consistently, overexpression of NFκB2 complex (p52‐RELB) dramatically triggered FAS expression and cell death (Figure 3J). Given NFκB2 complex binds to 3′‐2a region of *FAS* promoter in our EMSA assay, we propose that NFκB2 might be a transcriptional activator of FAS. In agreement with this proposal, overexpression of NFκB2 complex remarkably activated luciferase activity driven by full‐length *FAS* promoter, but not its Δ3′‐2a deletion mutant (Figure 3K). Moreover, there exists positive correlation between the expression levels of NFκB2 and FAS in either TCGA CRC dataset (Figure S3H, Supporting Information) and previous GEO CRC dataset (Figure S3I, Supporting Information). These observations reinforce our hypothesis that PIR suppresses FAS transcriptional activities possibly by interfering the binding of NFκB2 to *FAS* promoter.

### 2.4 PIR Inhibits NFκB2 Activation and FAS Membrane Translocation

**Figure 4:**
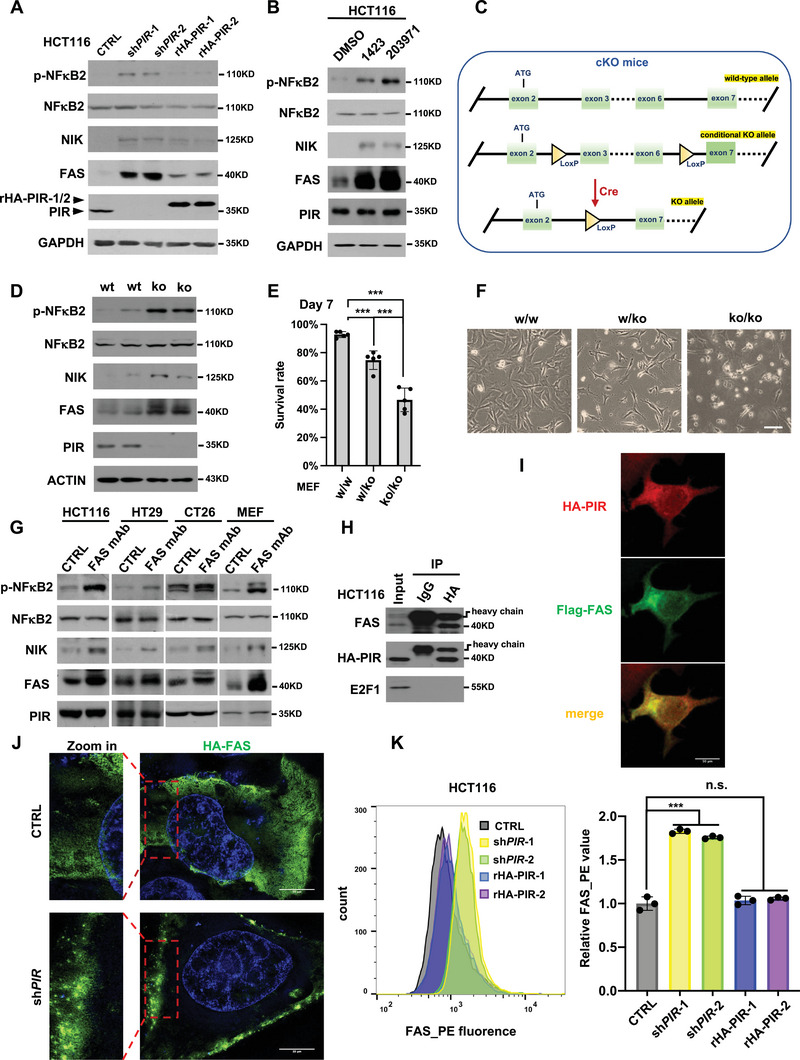
PIR inhibits FAS membrane translocation and NFκB2 activation. A) HCT116 cells w/wo pre‐rescuing expression of HA‐PIR were knocked down for PIR. 48 hours later, cells were detected for expression of indicated proteins. B) HCT116 cells were separately treated with 10 µm of PIR inhibitors CCG‐1423 and CCG‐203971 for 12 hours and then detected for indicated proteins. C) Simplified description of strategy for establishment of conditional PIR knockout (cKO) mice. D) PIR KO MEFs were created by expressing adenovirus‐based Cre recombinase in primary MEFs derived from PIR cKO mice, followed by detection of indicated proteins. E) PIR KO MEFs were generated and determined for survival rate by flow cytometry in the seventh day after Cre‐Ad administration. F) The morphology of the same PIR KO MEFs as in (E). G) HCT116, HT29, CT26, and MEF cells were treated w/wo anti‐FAS‐mAb (2.5 µg mL^−1^ for HCT116 and HT29 cells, 10 µg mL^−1^ for CT26 and MEF cells) for 24 hours, followed by detection of indicated proteins. H) HA‐PIR was transiently expressed in HCT116 cells and Co‐IP assays were performed with anti‐HA to determine the association of HA‐FAS with endogenous PIR. E2F1 was detected as a negative control. I) Immunofluorescence staining was performed to determine the co‐localization of Flag‐FAS (green) and HA‐PIR (red) in MEFs. Scale bars represent 50 µm. J). HA‐FAS were overexpressed in MEFs w/wo PIR KD, followed by immunofluorescence (IF) staining of FAS (green). Nuclei (blue) was stained with DAPI. Scale bar, 10 µm. K) HCT116 cells w/wo pre‐rescuing expression of HA‐PIR were knocked down for PIR. 48 hours later, cells were stained with anti‐FAS‐PE and determined for membrane‐distributed FAS with flow cytometry (left). The relative PE fluorescence intensities were shown as mean±SD. (n = 3, unpaired Student's *t*‐test, ***p < 0.01, n.s.: no significant difference) in the right.

It has been well clarified that NIK‐mediated NFκB2 activation (p100 phosphorylation and processing to p52) and subsequent translocation to nucleus is the prerequisite for its transcriptional activity. We thus examined whether PIR participate in the regulation of NFκB2 activation and translocation by NIK. PIR knockdown with sh*PIR* dramatically increased NIK protein level, activated NFκB2 (indicated by phosphorylation) and upregulated FAS protein level (**Figure** **4**A), and such alteration was reversed by re‐expression of HA‐tagged PIR in HCT116 cells (Figure 4A). Similar result was observed in HCT116 and HT29 cells when PIR was suppressed by its inhibitors CCG‐1423 and CCG‐203971 (Figure 4B; Figure S4A, Supporting Information). Consistently, PIR KD enhanced nuclear accumulation of NFκB2 in MEFs (Figure S4B, Supporting Information). Moreover, overexpression of Flag‐NIK dramatically promoted NFκB2 processing and translocation as well as FAS expression, and such effect was totally blocked by co‐expression of Flag‐PIR which successfully abolished NIK expression (Figure S4C, Supporting Information). These results demonstrate that PIR suppress NFκB2 activation and translocation probably via downregulating NIK protein level. We were then prompted to investigate the preliminary mechanism by which PIR regulates NIK. NIK protein level was dramatically decreased by co‐expression of PIR, and such effect was alleviated by MG132 treatment (Figure S4D, Supporting Information), indicating that PIR promotes NIK degradation at least partially through proteasome pathway. To confirm above observation, we generated *PIR‐LoxP/LoxP* conditional knockout mice (cKO) (Figure 4C), isolated MEFs and knocked out PIR in MEFs by expressing Cre recombinase. As expected, PIR KO significantly promoted the expression of NIK, p‐NFκB2 and FAS (Figure 4D), as well as the spontaneous death of MEFs (Figure 4E,F).

NIK integrates signals from a series of TNF receptor family members and sequentially activates p100 phosphorylation and processing, resulting in NFκB2 activation and translocation. Whether FAS can promote NIK‐NFκB2 pathway as a member of TNF receptor superfamily is largely unknown. To address this question, we treated HCT116, HT29, CT26 and MEF cells with FAS mAb, a commonly used activator of FAS death pathway by stimulating FAS trimerization. FAS mAb increased the levels of NIK, p‐NFκB2 and FAS (Figure 4G). Similarly, the protein levels of NIK and p‐NFκB2 were elevated by overexpression of FAS in a dose‐dependent manner (Figure S4E, Supporting Information). These results indicate that there may exist a positive feedback loop between FAS activation and NIK‐facilitated, NFκB2‐activated FAS expression (Figure S7A, Supporting Information), among which PIR acts as an important interrupter.

In a previous screening for PIR‐interacting proteins by virtue of immunoprecipitation of PIR followed by mass spectrometry analysis, we identified FAS as a candidate. The interaction between PIR and FAS was confirmed by co‐IP assay in HCT116 cells (Figure 4H). Consistently, immunostaining indicates a cytosolic co‐localization of these two molecules (Figure 4I). As aforementioned, FAS membrane translocation is a key event in FAS‐mediated apoptotic pathway. We then wondered if PIR influences membrane distribution of FAS. HA‐tagged FAS exhibited diffuse cytoplasmic distribution in normal condition, in contrast, it adopted a puncta‐like membrane distribution after PIR KD (Figure 4J). To confirm this observation, cells were stained with anti‐FAS‐PE and analyzed with flow cytometry for relative levels of membrane‐distributed FAS. PIR KD increased membrane‐distributed FAS and such effect was completely abolished by rescuing expression of PIR (Figure 4K). These observations suggest that cytosol‐localized PIR interacts with FAS and hence detains FAS in cytosol result in blockage of its membrane translocation. In summary, PIR inhibits FAS‐meditated cell death in at least three ways, namely, blockage of NIK‐mediated NFκB2 activation and NFκB2 association with *FAS* promoter to disrupt FAS transcriptional activities, and suppression of FAS membrane translocation.

### 2.5 PIR is Excessively Expressed in CRC and Its Inhibition Attenuates Cancer Formation

**Figure 5:**
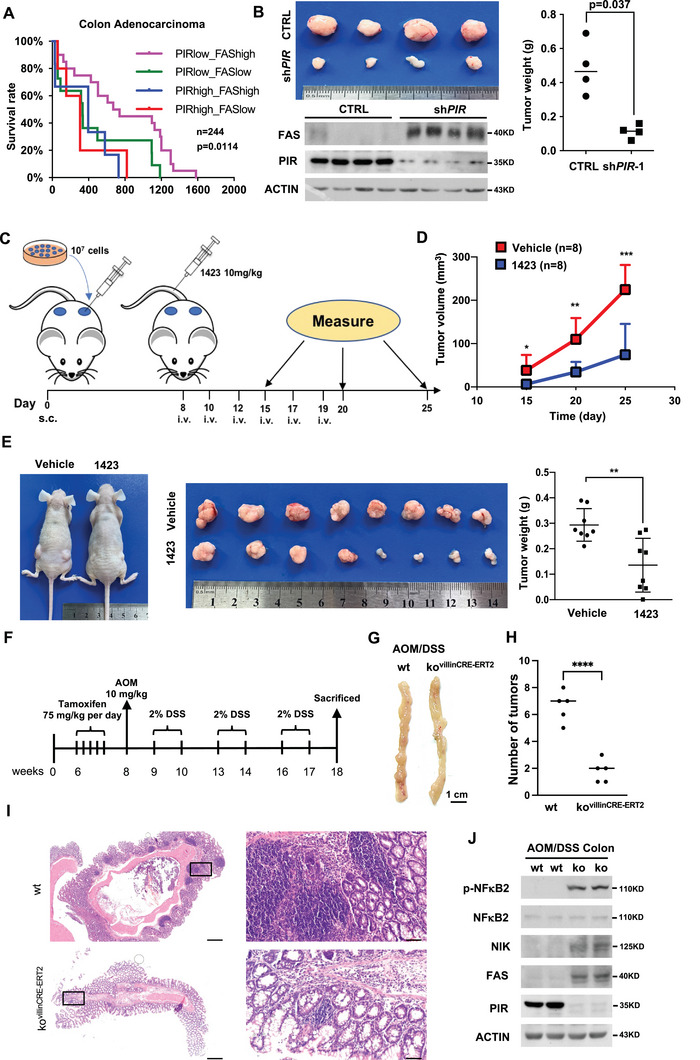
PIR inhibition attenuates colon cancer formation. A) A Kaplan‐Meier survival plot of patients with different expression of FAS and PIR in colon adenocarcinoma. Data are divided into 4 group based on mean expression of PIR and FAS as indicated. Data are publicly available from the Oncomine database. Statistical analysis was performed by the Log‐rank test. B) The nude mice were subcutaneously injected with wildtype (ctrl) and sh*PIR*‐1 HCT116 cells (10^6^ cells) on the left and right flanks, respectively, and observed for xenograft formation for 4 weeks. Dissected tumors (upper left) were weighed and analyzed employing unpaired Student's *t*‐test (right, n = 4). Indicated proteins in corresponding tumors were detected by WB (lower left). C) The Schematic diagram depicting the procedure of xenograft tumor treatment with PIR inhibitor CCG‐1423. In brief, 8 nude mice were subcutaneously injected with 10^7^ HCT116 cells on left and right flanks. Mice were randomly divided into two group. One week later, mice were intravenously injected with PBS (control group) or PIR inhibitor CCG‐1423 (10 mg kg^−1^ each time) three times a week for 2 weeks. Tumor volume was measured on day 15^th^, 20^th^ and 25^th^. s.c.: subcutaneous Injections; i.v.: intravenous Injections. D) Tumor volume of control group and CCG‐1423 treated group in (C)were shown as mean±SD and analyzed by using unpaired Student's *t*‐test (*p < 0.05, **p < 0.01, ***p < 0.001). E) The representative xenograft pictures of mice separately treated with Vehicle and CCG‐1423 (left panel). Dissected tumors (middle) were weighed and analyzed with students’ *t* test (right panel, n = 8, **p < 0.01). F) A simplified experimental design of AOM/DSS induced colon cancer mice model. G and H) Representative photographs (G) of the colons form the animals in wild‐type and PIR‐knockout mice. Tumor numbers were calculated and analyzed with students’ *t* test (H, n = 5, ****p < 0.0001). I) Representative photographs of the histopathology of the colon tissue. Scale bars, 500 µm for low magnification (1 ×, left panel), and 50 µm for high magnification (10 ×, right panel). J) WB was performed to determine the indicated protein levels in wild‐type or PIR‐knockout colon tissues.

After clarifying the regulatory mechanism of PIR against FAS‐mediated cell death, we turned to figure out PIR expression profile and the correlation between PIR and NIK‐NFκB2‐FAS pathway in CRC. PIR was significantly upregulated in CRC as compared with corresponding normal tissues according to public TCGA and GEO database (Figure S1A, Supporting Information), while FAS is downregulated in colorectal cancer (Figure S2G, Supporting Information). Similar result was obtained in human CRC samples by WB analysis (Figure S5A, Supporting Information). Additionally, we divided human CRC samples into PIR‐Low group and PIR‐High group according to PIR expression, and found that FAS predominantly displayed a membrane‐associated distribution in PIR‐Low group, in contrast, a dispersed cytosolic distribution in PIR‐High group (Figure S5B, Supporting Information). Moreover, PIR was highly expressed in colon cancer cell lines and rarely expressed in normal cell lines MEF and HFF (Figure S5C, Supporting Information). Furthermore, FAS is negatively correlated with PIR (Figure S2G, Supporting Information), and positively correlated with NFκB2 (Figure S3H, Supporting Information) and NIK (Figure S5D, Supporting Information) in 54 paired colon cancer patient samples. Moreover, we analyzed a set of colon cancer cell lines from GEO database (GSE28567^[^ ^20^ ^]^) and also found negative correlation of FAS with PIR and positive correlation with NFκB2 and NIK (Figure S5E, Supporting Information). In addition, lower expression of PIR plus higher expression of FAS is correlated with the highest survival rate in colon adenocarcinoma dataset derived from Oncomine database (**Figure** **5**A). These results suggest that PIR may facilitate tumor survival by inhibiting FAS‐NIK‐NFκB2‐FAS positive feedback loop of FAS expression and FAS‐mediated cell death in colon cancers. To confirm this proposal, we performed xenograft tumor formation assays with HCT116 cells and found that PIR KD dramatically retarded tumor formation and upregulated FAS expression accordingly (Figure 5B). Consistently, intravenous administration of PIR inhibitor CCG‐1423 also effectively inhibited tumor formation (Figure 5C–E). To verify the findings that PIR promotes CRC development genetically, we constructed tamoxifen‐induced colon submucosa‐specific PIR‐knockout mouse. The role of PIR in colon cancer development was then investigated in a well‐established colitis‐associated colorectal tumor model. A simplified working procedure was drawn in Figure 5F. We found that the sizes and numbers of colon tumors in PIR‐knockout mice were remarkably lower than in wild‐type mice (Figure 5G and H). This observation was confirmed by H&E staining of the tumors in colons (Figure 5I). Consistently, Western blot analysis showed an increase expression of p‐NFκB2, NIK, and FAS in the colon tumors of PIR‐knockout mice as compared with wild‐type mice (Figure 5J). It is worth noting that the inhibitory effect of PIR inhibitors on cell survival is dependent on PIR expression level, as all of three inhibitors were able to dramatically induce cell death of HCT116, HT29 and SW620 with high expression of PIR, but failed to influence the survival of HFF with very low expression of PIR (Figure S5F, Supporting Information). This observation indicates that PIR may be an emerging and promising target for therapy of colon cancer with high expression level of it.

### 2.6 PIR inhibition Sensitizes Cancer Cells to FAS‐Based Immunotherapy

**Figure 6:**
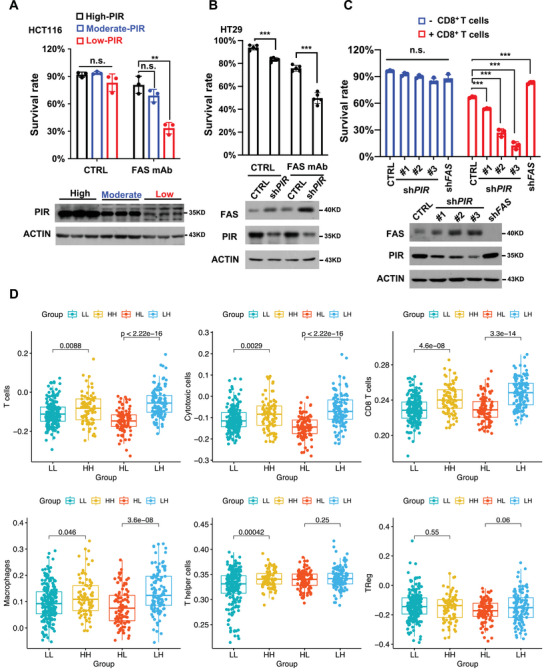
PIR inhibition sensitizes cells to FAS‐based immunotherapy. A) HCT116 cells were infected with blank vector viruses or different volume of sh*PIR*‐1 viruses. After 24 hours of infection, cells were treated with anti‐FAS‐mAb (2.5 µg mL^−1^) for 24 hours. Then survival rates were determined by PI staining. Data represent mean±SD. (n = 3, **p < 0.01, n.s.: no significant difference, unpaired Student's *t*‐test). PIR protein levels are shown in lower panel. B) HT29 cells were administrated with blank vector viruses (CTRL) or PIR KD viruses. After 24 hours of infection, cells were treated with anti‐FAS‐mAb (2.5 µg mL^−1^) for 24 hours, followed by detection of survival rate (n = 5, unpaired Student's *t*‐test, ***p < 0.001). C) HCT116 cell lines knocked down for FAS or different levels of PIR were created, labelled with GFP and incubated with pre‐activated human effector CD8^+^ T cells for 20 hours. Survival rates of HCT116 cells were determined employing flow cytometry. Bar diagram (upper panel) represents mean±SD. of three independent experiments (unpaired Student's *t*‐test, ***p < 0.001, n.s. no significance). PIR and FAS proteins were detected by WB (lower panel). D) Boxplot of various immune cells infiltration levels among tumors with different PIR and FAS expression status in colon cancer. *P* value were analyzed using Student's *t*‐test. Data were publicly available in TCGA database. HH: PIR‐high and FAS‐high expression; HL: PIR‐high and FAS‐low expression; LH: PIR‐low and FAS‐high expression; LL: PIR‐low and FAS‐low expression.

To further confirm inhibitory role of PIR in FAS‐mediated cell death, HCT116 cells expressing high, moderate and low level of PIR were generated by administrating cells with different titers of sh*PIR* viruses, and then treated with anti‐FAS mAb. As expected, decreased expression of PIR sensitizes cells to FAS‐mAb induced cell death (**Figure** **6**A). Consistently, PIR KD or KO dramatically accelerates cell death triggered by FAS‐mAb (Figure 6B ; Figure S6A, Supporting Information). Potentiation of the tumor‐killing ability of CD8^+^ T cells, along with their efficient tumor infiltration, is a key element of successful immunotherapies. Several studies have indicated that tumor cells escape CD8^+^ (cytotoxic) T cells in tumor microenvironment by decreasing their own FAS expression or acquiring lose‐of‐function mutations of components in FAS death pathway.^[^ ^21^ ^]^ To this end, we turn to explore whether PIR functions in cancer cells in avoiding immunosurveillance. By virtue of an in vitro assay system based on published method,^[^ ^22^ ^]^ we evaluated the tumor‐killing ability of human CD8^+^ T cells to HCT116 cells expressing different levels of PIR. HCT116 cells were co‐cultured with pre‐activated CD8^+^ T cells for 20 hours and then subjected to cell survival rate analysis. As expected, decreased expression of PIR dramatically sensitized HCT116 cells to CD8^+^ T cells‐induced cell death by upregulating FAS protein level and such effect was completely blocked by knockdown of FAS (Figure 6C; Figure S6B, Supporting Information). This functional assay reinforces our proposal that excessive expression of PIR may be a crucial event in tumorigenesis by conferring tumor cells resistance to immune clearance, suggesting that PIR is an emerging target for cancer immune therapy. The evidence presented above revealed the need to take the tumor microenvironmental immune parameters into account. As reported, T cells, especially cytotoxic T lymphocytes immune infiltration of human colorectal cancer is associated with favorable clinical outcome.^[^ ^23^ ^]^ To understand the immune infiltration in colorectal cancer based on PIR and FAS expression, we used TCGA‐COAD data to compare the transcriptional profile of the immune cell subpopulations in human colorectal cancer. T cells, cytotoxic lymphocytes, CD8^+^ T cells and macrophages, rather than T helper cells and Treg cells, were decreased in PIR high‐expression and FAS low‐expression tumors (Figure 6D). This investigation reveals a suppressive immune landscape in PIR‐high and FAS‐low expression human colon cancer.

**Figure 7:**
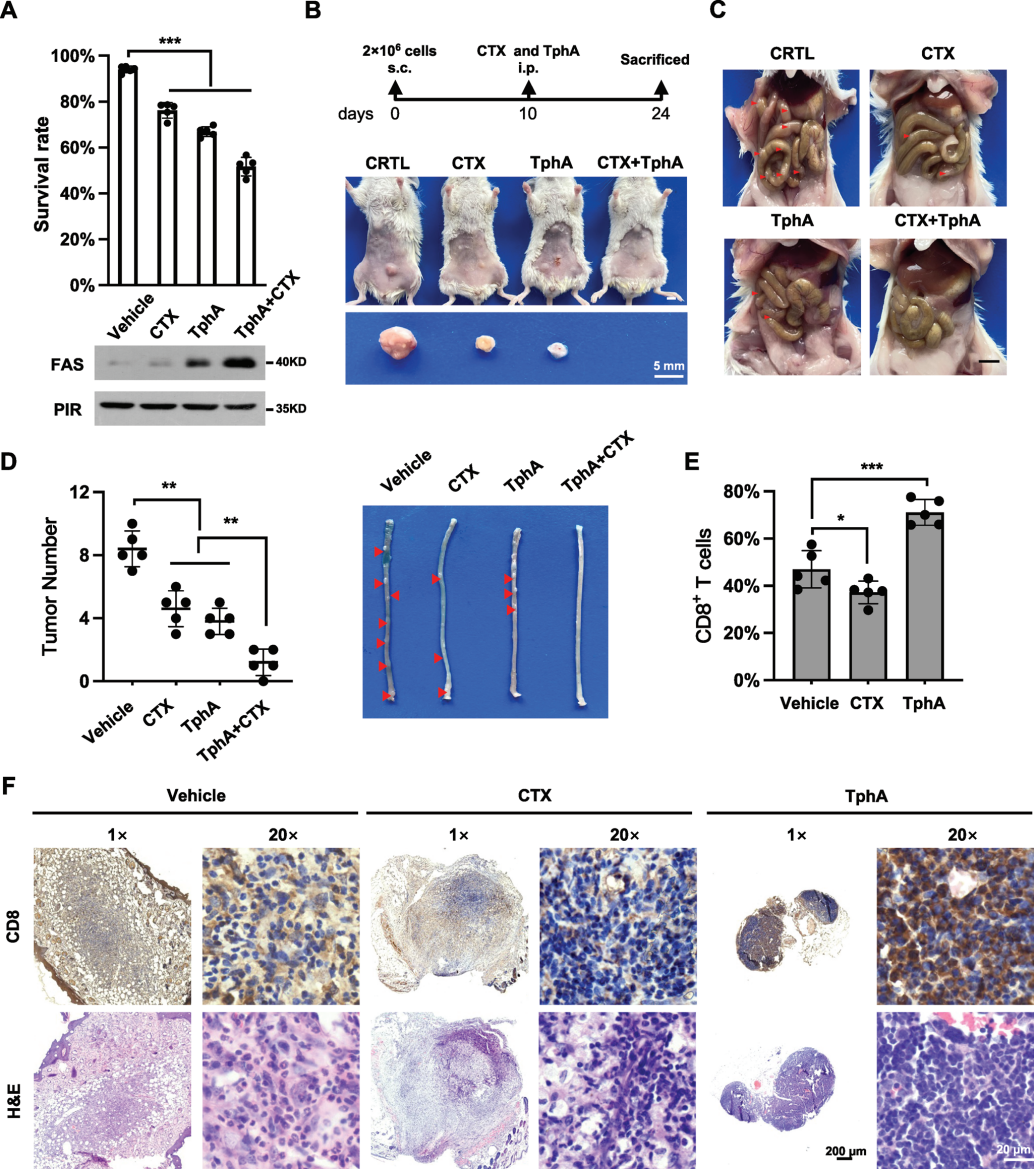
PIR suppresses FAS‐dependent apoptosis by switching off NF‐κB2‐FAS axis. A) CT26 cells were treated with cetuximab (CTX, 10 µg mL^−1^) and TphA (50 µg mL^−1^) alone or in combination for 24 hours, followed by detection of survival rate (upper panel, n = 5, unpaired Student's *t*‐test, ***p < 0.001) and FAS protein level (lower panel). B and C) Allograft tumors assays based on mouse CT26 cells were conducted according to the procedure indicated in upper panel of (B). Mice were sacrificed at day 24^th^. The representative in situ tumors (B) and metastatic intestinal tumors (C) from mice treated with CTX and TphA alone or in combination were shown. s.c.: subcutaneous injections; i.p.: intraperitoneal injections. Scale bars represent 5 mm. D) Colon tumor numbers (left panel) and tumor‐bearing colons (right panel) of the same mice in (B) and (C) were presented (n = 5, unpaired Student's *t*‐test, **p < 0.01). E and F) Quantitative analysis of the percentage of CD8^+^ T cells (E, n = 5, unpaired Student's *t*‐test, *p < 0.05, ***p < 0.001). IHC was performed with CD8^+^ T cell specific antibody to determine CD8^+^ T cell infiltration in situ tumors (F). Scale bars represent 200 and 20 µm separately.

To investigate whether inhibition of PIR enhances the therapeutic effect of cetuximab (EGFR mAb, a standard approach in clinical CRC treatment) on CRC, we created CT26 cell‐based allograft model in BALB/c mice. Both CT26 cells cultured in vitro and their allograft mice were treated with a combination of cetuximab and PIR inhibitor TphA. Surprisingly, CRC advancement was dramatically blocked by such combined treatment as compared with the treatment with either cetuximab or TphA alone (Figure 7A–D). Moreover, we observed a significant increase in the percentage of CD8^+^ T cells infiltration in CT26 allograft tumors treated with TphA rather than cetuximab (Figure 7E,F). These results suggest that PIR inhibitors strengthen the efficacy of cetuximab in CRC treatment possibly by triggering the anti‐tumor immunity of CD8^+^ T cells.

## 3 Conclusion

FASL and FAS have long been considered as a death system that plays an important role in the immune elimination of cancer cells.^[^ ^24^ ^]^ IL‐2 activated CD8^+^ T lymphocytes act as the main executor in eliminating cancer cells via initiation of FASL/FAS apoptosis pathway.^[^ ^25^ ^]^ Certain amount of endogenous FAS protein expression and subsequent translocation to membrane in cancer cells are the determinants for CD8^+^ T lymphocytes execution of FASL‐triggered apoptosis.^[^ ^26^ ^]^ In this study, we report a previously undefined role of PIR protein in protecting cancer cells from CD8^+^ Killer T cells executed apoptosis via multiple aspects of mechanism including disruption of FAS transcriptional activities and blockage of FAS membrane translocation (Figure S7A, Supporting Information). The key point of our study is the importance of endogenous FAS protein expression and its translocation to the cell membrane for CD8^+^ T lymphocyte‐mediated apoptosis. As to the regulation of FAS expression, we found that it is mature NFκB2 complex (p52‐RELB) rather than NFκB1 complex (p50‐RELA) that governs FAS expression by directly binding to its promoter region and activating its transcription in CRC. This finding is different from a previous report showing that canonical NFκB1, but not NFκB2, could promote FAS transcriptional activities.^[^ ^27^ ^]^ Such inconsistence may be attributed to the distinct cell lines and experimental condition used by two studies. Based on this notion that NFκB2 is a robust transcriptional activator of FAS, we further identified PIR as a strong suppressor against NFκB2‐mediate FAS transcriptional activities. PIR inhibits NFκB2 in two ways. On the one hand, PIR promotes proteasomal degradation of NIK, an upstream kinase of NFκB2 serving to integrate signals from TNF receptor family members and activate NFκB2 maturation (process of immature p100 to mature p52) and nuclear translocation, resulting in the interruption of NFκB2‐activating process. On the other hand, PIR interacts with RELB and disrupts the interaction of NFκB2 complex (p52‐RELB) with *FAS* promoter, leading to the blockage of NFκB2 transcriptional activity toward FAS. In these ways PIR dramatically inhibits FAS expression. In addition, PIR interacts with FAS and detains it in cytosol, preventing its membrane translocation, an event required for its subsequent trimerization and activation. It is important to point out that FAS mAb‐triggered FAS activation could also induce NIK and FAS expression, establishing a positive feedback loop between FAS activation and NIK‐facilitated NFκB2‐mediated FAS expression. Such auto amplification of FAS signal is supposed to be disrupted by excessive expression of PIR. Taking together, we establish a PIR‐NIK‐NFκB2‐FAS survival signaling transduction pathway and suggest that PIR is an important survival factor functioning to promote CRC malignancy by inhibiting FAS‐based apoptosis in multiple mechanisms. It is important to note that we primarily focus on CRC in this study and are actually unclear whether these mechanisms are CRC‐selective or CRC‐specific up to now. To clarify this issue, other cancer types should be analyzed in the future.

In this study we also provide convincing evidence proving that PIR may be an emerging target for therapy of CRC. First, endogenous FAS upregulation resulted from PIR KD or suppression of PIR with its inhibitors can significantly trigger apoptosis even without stimulation of upstream initiators such as FAS ligand or FAS mAb. This observation is confirmed by colon specific PIR knockout mice that show high level of FAS expression and dramatically retard AOM/DSS‐induced colon cancer formation. Second, wildtype HCT116 cells or HT29 cells with high expression of PIR are resistant to FAS mAb‐ or CD8^+^ T cells‐induced cell death, in contrast, moderate downregulation of PIR even though being not sufficient to initiate apoptosis can dramatically sensitize HCT116 colorectal cancer cells to either FAS mAb‐ or CD8^+^ T cells‐induced cell death. Third, administration of PIR inhibitor can significantly retard mice xenograft tumor growth of HCT116 cells. Taken together, inhibition of PIR may be a promising strategy for therapy of CRC with high level of PIR and low level of FAS. This suggestion is well supported by statistics from large amounts of clinical data that reveal a negative correlation of PIR expression with NIK and FAS expression, as well as with the survival probability. Consistently, it was reported that NIK activity is required for T cell antitumor immunity.^[^ ^28^ ^]^

In recent years, blockade of immune checkpoint such as PD‐1/PD‐L1 has exhibited overwhelming success in the therapy of some kinds of cancers.^[^ ^29^ ^]^ However, only a minority of patients responded well in the clinical trials.^[^ ^30^ ^]^ It is reasonable that tumor cells with very low level of endogenous FAS expression may be still resistant to active CD8^+^ killer T cell‐executed death. Indeed, it is reported that disfunction of FAS‐mediated apoptosis pathway contributing to failure of immunotherapy in CRC.^[^ ^31^ ^]^ Based on our evidences that PIR expression is negatively correlated with FAS‐initiated cell death, we believe that PIR‐caused silence of endogenous FAS expression in tumor cells may weaken the function of active CD8^+^ killer T cells in tumor microenvironment and be the potential reason leading to tumor resistance to immune checkpoint inhibitors. Notably, CRC exhibits heterogeneity and is classified into several consensus molecular subtype (CMS), each with different responses to immunotherapy.^[^ ^32^ ^]^ CMS1 and CMS4 are characterized by high levels of immune infiltration. The CRC cell lines used in this study mainly represent CMS1 (HCT116 and HT29) and CMS4 (CT26), which are considered as “hot” tumor with a favorable response to immunotherapy. According to our finding that PIR inhibitors can enhance anti‐tumor activity of CD8^+^ killer T cells by stimulating endogenous FAS expression and its membrane translocation in tumor, combination of PIR inhibitors and immune checkpoint blockade may be a potential strategy to the treatment of CRC, particularly those resistant to immunotherapy. It is therefore urgent to develop more potent PIR inhibitors in the future.

## 4 Experimental Section

### Patients and Colon Tissue Specimen

CRC of 32 pairs and adjacent tissue paraffin sections (ZL‐ColA961) were purchased from Shanghai Well Biotech (Shanghai, China). Four pairs of CRC specimens and adjacent normal colon tissues used for immunochemical analysis were obtained from the First Affiliated Hospital of Xiamen University (Xiamen, China) with patient consent and institutional review board approval. The study protocol conformed to the ethical guidelines and was approved by the Institute Research Ethics Committee at Xiamen University.

### Cell Lines

HEK293T, HCT116, HT29, MEF, HFF, and SW620 cells were taken from the laboratory cells bank. CT26 cells were obtained from Xiamen Immocell Biotechnology Co., Ltd (IML‐026, China). All cell lines were cultured in Dulbecco's modified Eagle's medium (DMEM) with 10% fetal bovine serum (FBS) at 37 °C in 5% CO_2_ (v/v). All cell lines were tested as free for mycoplasma contamination. HCT116 cells used in this manuscript were characterized by Guangzhou Cellcook Biotech Co., Ltd (Guangzhou, China) using short tandem repeat (STR) markers.

MEFs (mouse embryonic fibroblasts) from WT and PIR KO mice were isolated and cultured in DMEM. Briefly, primary MEFs were isolated from E13.5 embryo. Heads were used to identify genotype of the embryo, while the rest of the embryo (viscera and limbs excluded) was dissociated with 1 mL of 0.05% trypsin in an Eppendorf tube for 30 min at 37  °C, pipetted for complete dissociation, and incubated for another 5 min at 37  °C. Dissociated cells were then supplemented with 2 mL of complete medium, centrifuged for 5 min at 150 × *g* to discard the trypsin, and finally seeded on gelatin‐coated (0.1%) ø 10 cm plates.^[^ ^33^ ^]^

### Antibodies and Reagents

**Table:** 

REAGENT or RESOURCE	SOURCE	IDENTIFIER
**Antibodies**		
PIR (D‐12)	Santa Cruz	Cat# sc‐271622 RRID: AB_10709292
HA (F‐7)	Santa Cruz	Cat# sc‐7392 RRID: AB_627809
FAS	Proteintech	Cat# 13098‐1‐AP RRID: AB_2278042
Cytochrome c	Proteintech	Cat# 66264‐1‐Ig RRID: AB_2716798
COXIV	Proteintech	Cat# 11242‐1‐AP RRID: AB_2085278
GAPDH	Proteintech	Cat# 60004‐1‐Ig RRID: AB_2107436
Lamin B1	Proteintech	Cat# 66095‐1‐Ig RRID: AB_11232208
TRAF3	Proteintech	Cat# 66093‐1‐Ig RRID: AB_10837364
Caspase 8	Proteintech	Cat# 66093‐1‐Ig RRID: AB_11232214
CD8 mouse McAb	Proteintech	Cat# 66868‐1‐Ig RRID: AB_2882205
Cleaved Caspase3	Cell Signaling Technology	Cat# 9661S RRID: AB_2341188
Caspase 3	Cell Signaling Technology	Cat# 9665S RRID: AB_10698879
NFκB1	Cell Signaling Technology	Cat# 13586S RRID: AB_2665516
NIK (4A2)	Cell Signaling Technology	Cat# 4994S RRID: AB_2297422
HA (Rabbit)	Cell Signaling Technology	Cat# 3724S RRID: AB_1549585
p‐NFκB2 (Ser866/870)	Cell Signaling Technology	Cat# 4810S RRID: AB_659925
NFκB2	Cell Signaling Technology	Cat# 37359S RRID: AB_2799114
Flag	SIGMA	Cat# F1804 RRID: AB_262044
PIR (for IHC only)	SIGMA	Cat# HPA000697 RRID: AB_627809
Anti‐FLAG M2 beads	SIGMA	Cat#A2220 RRID: AB_10063035
actin	SIGMA	Cat#A1978; RRID: AB_476692
hFAS (human, activating)	Millipore	Cat#05‐201; RRID: AB_309653
mFAS (mouse, activating)	Millipore	Cat#554 254; RRID: AB_395326
Anti‐human CD3	Biolegend	Cat# 317 325 RRID: AB_11147370
Anti‐human CD28	Biolegend	Cat# 302 933 RRID: AB_11150591
FAS_PE	eBioscience	Cat#12‐0959‐42; RRID: AB_10853323
**Bacterial and Virus Strains**		
*Ecoli*.BL21(DE3) competent cell	Lab preserve	N/A
**Chemicals, Peptides, and Recombinant Proteins**		
CCG‐203971	Selleck Chemicals	S8469
CCG‐1423	Selleck Chemicals	S7719
Z‐VAD‐FMK	Selleck Chemicals	S7023
MG132	Selleck Chemicals	S2619
Triphenyl Compound A (TphA)	Santa Cruz	sc‐364144A
PEI MAX	Polysciences	Cat#24765
human IL‐2	Biolegend	Cat#589102
Lymphopure™	Biolegend	Cat#426201
MojoSort™ Human CD8 Naïve T Cell Isolation Kit	Biolegend	Cat#480045
human Fas Ligand	Cell Signaling Technology	Cat#5452SF
HisPur Ni‐NTA spin columns	ThermoFisher Scientific	Cat#88226
Turbofect	Life Technology	Cat#R0532
**Critical Commercial Assays**		
FITC Annexin V Apoptosis Detection Kit	BD PharmingenTM	Cat#556547
**Experimental Models: Cell Lines**		
HCT116 (human)	Lab preserve	N/A
SW620 (human)	Lab preserve	N/A
HEK293T (human)	Lab preserve	N/A
MEF (mouse)	Lab preserve	N/A
HT29 (human)	Lab preserve	N/A
HFF (human)	Lab preserve	N/A
**Experimental Models: Organisms/Strains**		
BALB/cJ	Jackson Laboratory	Cat# 000651
Oligonucleotides		
sh*PIR*‐1 targeting sequence: 5′‐GAAGCCACTTTGTCTTAATT‐3′	This paper	N/A
sh*PIR*‐2 targeting sequence: 5′‐GAACACCAATGAAGAGATTT‐3′	This paper	N/A
sh*PIR*‐5 targeting sequence: 5′‐GAAGTCAAAGATTGGAAACTA‐3′	This paper	N/A
sh*NFκB2* targeting sequence: 5′‐AGCAAGCCAGCCTCGGCCGA‐3′	This paper	N/A
sh*FAS* targeting sequence: 5′‐AGCAAGCCAGCCTCGGCCGA −3′	This paper	N/A
sh*mFAS* targeting sequence: 5′‐CCTCAAATCTTAGCTTGAGTA‐3′	This paper	N/A
sh*DDIT3* targeting sequence: 5′‐AGCAAGCCAGCCTCGGCCGA −3′	This paper	N/A
sh*mDDIT3* targeting sequence: 5′‐AGGAAGAACTAGGAAACGGAA‐3′	This paper	N/A
*Fas*‐3′−2a probe: 5′‐AACGTCTGTGAGCCTCTCATGTTGCAGCCA −3′	This paper	N/A
**Software and Algorithms**		
ImageJ	Image J	RRID:SCR_001935
Prism 8	GraphPad	RRID: SCR_0 02798
RStudio	RStudio	RRID: SCR_000432
FlowJo	FlowJo	RRID: SCR_008520
SnapGene	SnapGene	RRID: SCR_01 5052

### Animal Studies

The nude mice (BALB/cJ) used in this study were obtained from Animal Center of Xiamen University. C57BL/6 mice (*PIR‐flox*, strain NO. T025465) were generated commercially by GemPharmatech Co., Ltd. All animals were housed with 12 h light/darkness and standard chow diet at 25 °C. 6‐8‐week‐old male mice were used for xenograft assays. In all animal studies, mice were randomly allocated to the experimental groups.

For xenograft studies, 1 × 10^6^ cells were injected subcutaneously into both flanks of 6‐week‐old male nude mice. Tumor size were monitored weekly using a digital caliper. After 4 weeks of injection mice were sacrificed by carbon dioxide asphyxia and xenograft tumors were dissected and measured for weight.

For in vivo drug treatment assays, HCT116 cells (10^7^ cells) were injected subcutaneously into both flanks of 6‐week‐old male nude mice. On the eighth day after cell injection, PIR inhibitor CCG‐1423 and vehicle were injected intravenously (i.v.) into randomly grouped mice, three times a week. Tumor size was measured on 15^th^, 20^th^ and 25^th^ days with a digital caliper. On 25^th^ day all mice were sacrificed by carbon dioxide asphyxia and xenograft tumors were dissected and measured for weight.

For Cetuximab and TphA administration assays, CT26 cells (2 × 10^6^ cells) were injected subcutaneously into BALB/c mice. On the tenth day after cell implantation, standard CRC treatment drug Cetuximab (2 mg kg^−1^), PIR inhibitor TphA (15 mg kg^−1^) or vehicle were administrated intraperitoneal (i.p.) into randomly grouped mice. Metastatic intestinal tumor number were measured on 24^th^ days.

Colitis‐associated colon tumor formation (AOM/DSS induced colon cancer mice model) was induced in mice as previously reported.^[^ ^34^ ^]^ Briefly, 6‐week‐old male mice were injected intraperitoneally tamoxifen(75 mg kg^−1^) per day for five days. Two weeks later, mice were injected intraperitoneally with 10 mg kg^−1^ azoxymethane (AOM) (A5486; Sigma‐Aldrich). Seven days after AOM injection, these mice were provided with 2% dextran sodium sulfate (DSS) (MP Biomedicals) in their drinking water for 7 consecutive days, followed by another 14 days of recovery. This round was repeated twice until 18 weeks, when all mice were sacrificed.

### Construction of Plasmids

Full‐length cDNAs encoding human *PIR* (gene ID: 8544), *NFκB2* (gene ID: 4791), *FAS* (gene ID: 355), and *RELB* (gene ID: 5971) was amplified from human cDNA. Point mutations were created by a PCR‐based site‐directed mutagenesis method. Expression plasmids for various proteins were constructed into the pcDNA3.3 and pLV‐EF1a vector for transfection or in pBoBi vectors for lentivirus infection. shRNA plasmids targeting human genome *PIR*, *DDIT3*, *FAS* and *NFκB2* were constructed into lentivirus‐based vector pLKO.1.

### Plasmid Transfection and Virus Infection

Expression constructs were transfected into HEK293T according to a previously described method.^[^ ^35^ ^]^ Briefly, lentivirus was produced by co‐transfecting HEK293T cells with VSVG, Rev, pMDL and suggested constructs. Virus was collected 48 hours after transfection. Virus infection was carried out by incubating cells with corresponding virus and polybrene(10 µg mL^−1^)for 24 hours. The infected cells were selected with puromycin for 24 hours (mRNA analysis) or 48 hours (protein and phenotypic analyses).

### Immunoprecipitation (IP) and Western Blot (WB) Analysis

IP and WB experiments were conducted as previously described.^[^ ^35^ ^]^ Briefly, cells were lysed with cell lysis buffer and sonicated twelve times for 1 sec each, and centrifuged at 15,000 *g* for 30 min at 4 °C to obtain supernatant as total cell lysate. For IP, total cell lysate was pre‐cleared with 5 µL protein A/G beads for 1 h, and then incubated with 1 µg protein A/G beads‐bound isotype‐matched IgG control or indicated antibodies for another 3 hours. The immunoprecipitants were collected by centrifugation and then resolved by SDS‐PAGE.

### Immunofluorescence (IF)

Immunofluorescence were conducted as previously described.^[^ ^35^ ^]^ Cells were cultured on glass coverslips in 6‐well plates at 30%−40% of confluence and fixed with 40% paraformaldehyde for 10 min, followed by permeabilization with 0.2% Triton X‐100 in PBS for 10 min at room temperature. After two times washes in washing buffer, the coverslips were blocked with blocking solution for 1 hour and then incubated with corresponding primary antibodies at 4 °C overnight. Next, cells were rinsed four times (5 minutes each) with washing buffer, followed by incubation with appropriate secondary antibodies conjugated with Alexa Fluorescence 488 or 555 in blocking solution for 1 hour at 37 °C, in dark. Finally, the slides were stained with DAPI(1 µg mL^−1^) in washing solution for 2 minutes, washed for four times, mounted with 90% glycerol and sealed with nail polish. Images were captured using a Zeiss Laser Scanning Microscope (LSM) 880 at pixels of 1024 × 1024.

### Reverse Transcription and Quantitative PCR (RT‐qPCR)

cDNA was prepared from 500 ng RNA with the SuperScript Reverse Transcriptase II kit (Invitrogen) using oligo dT or random hexamer primers. The primer sequences used for qPCR were as follows:

hPIR, 5′‐GTGGAGCCTCAGTACCAGGA‐3′ and 5′‐AAATGGACCATGTTGGATAACTGG‐3′;
hFAS: 5′‐TGACCCTTGCACCAAATGTGA‐3′ and 5′‐AGAAGACAAAGCCACCCCAA‐3′;
hDDIT3: 5′‐CCTCCTGGAAATGAAGAGGAAGA‐3′ and 5′‐TCCTGGTTCTCCCTTGGTCT‐3′;
hGAPDH: 5′ ‐GCTCTCTGCTCCTCCTGTTC‐3′ and 5′‐GCTCTCTGCTCCTCCTGTTC‐3′;
hTNFSRF10B: 5′‐CCACAAAGAATCAGGTACAAA −3′ and 5′‐GGCTCGGATCATCTCTGCTC −3′;
mPIR, 5′‐GCGATGGATATTCAGATGTTA‐3′ and 5′‐TAACATCTGAATATCCATCGC −3′;
mFAS: 5′‐GTGTTCTCTTTGCCAGCAAAT −3′ and 5′‐ATTTGCTGGCAAAGAGAACAC −3′;
mDDIT3: 5′‐AGGAAGAACTAGGAAACGGAA −3′ and 5′‐TTCCGTTTCCTAGTTCTTCCT −3′;
mGAPDH: 5′‐GCTCTCTGCTCCTCCTGTTC −3′ and 5′‐TTTGCCACTGCAAATGGCAGC −3′;
mTNFSRF10B: 5′‐CCACAAAGAATCAGGTACAAA −3′ and 5′‐ATTCTCATAATACTGAAGAGC −3′.

The forward and reverse primers were used at a final concentration of 400 nM in 1 × SYBR Green Supermix (Promega). The thermocycling program was 10 min at 95 °C to denature the cDNA, followed by 40 cycles of 30s at 95 °C, 30s at 55—60 °C and 30s at 72 °C, and another 95 °C denaturing step for 15s prior to a melting curve sequence from 65 °C to 95 °C in 0.5 °C increments. The program was performed using a CFX96 Real‐Time PCR Detection System on a C1000 Thermocycler (Bio‐Rad) and analyzed using the Bio‐Rad CFX Manager software (v.2.0). The threshold cycles (Ct) were normalized to reference gene GAPDH. The identity of the amplicon was confirmed by melting curve data. Appropriate no‐template and no‐reverse transcriptase negative controls were included in RT–qPCR running of each gene.

### JC‐1 Assay

Cells were washed in PBS and incubated in JC‐1 staining reagent (Invitrogen) at 37 °C for 15 min. After staining the cells were successively washed once in assay buffer, maintained in PBS, and analyzed by flow cytometry.

### Chromatin Immunoprecipitation (ChIP) Assays

ChIP assays were performed as follow. Total 10 × 10^7^ cells were pre‐treated as described and crosslinked for 15 min at room temperature using 1% formaldehyde. The crosslinking was terminated by adding glycine with a final concentration of 0.125 m for 5 min. Nuclei were extracted by Mg‐Ni‐NP40 buffer [Tris‐HCl (15 mm) at pH7.5, MgCl_2_ (5 mm), KCl (45 mm), DTT (0. 5 mm), NaCl (15 mm), sucrose (0.3 mm) and 0.01% NP40] and lysed by lysis buffer [Tris‐HCL (50 mm) at pH8.0, EDTA (2 mm), 1% sodium dodecyl sulfate and cocktail]. The chromatin was then sheared by sonication on ice. Lysates were incubated with antibody or normal mouse IgG overnight at 4 °C, and then protein was digested using proteinase K. The ChIP enriched DNA was subjected to PCR using the following primers: FAS‐ChIP‐F: 5′‐GCTGGGGCTATGCGATTTGGC‐3′ and FAS‐ChIP‐R: 5′‐TGGCTGCAACATGAGAGGCTCACAGACGTT‐3′.

### Next‐Generation RNA Sequencing

HCT116 cells were infected with lentiviruses packaged from control or *PIR* shRNA vector and maintained in complete culture medium for 48 days. Then total RNA was extracted using the Trizol‐based method (Takara), evaluated by gel electrophoresis for the integrity and quality and measured for concentration using a Nano Drop 2000 (Thermo Fisher Scientific). To construct mRNA library, purified mRNA was fragmented into small pieces, followed by synthesis of cDNA using random hexamer‐primed reverse transcription. Afterward, the cDNA fragments were amplified by PCR, and subjected to end repair by incubating with Mix and RNA Index Adapters. Next the repaired cDNA fragments were amplified by PCR again, and PCR products were purified by AMPure XP Beads and validated on the Agilent Technologies 2100 bioanalyzer for quality control. The double stranded PCR products from previous step were heat denatured and circularized by the splint oligo sequence to get the final library. The single strand circle DNA (ssCir DNA) was formatted as the final library and amplified with phi29 to make DNA nanoball (DNB) which had more than 300 copies for every molecule. Finally, DNBs were loaded into the patterned nanoarray and single‐end 50‐base reads were generated on BGIseq500 platform (BGI‐Shenzhen, China).

### Survival Rate Analysis with Flow Cytometry

For survival rate analysis in majority of experiments, cells were trypsinized, washed with PBS, incubated with both FITC‐Annexin V and PI or PI alone for 15 min, and then subjected to flow cytometry to analyze survival rate by gating cells with lower fluorescence of Annexin V and PI staining.

To analyze the survival rate of HeLa cells treated with activated CD8^+^ T cells, HeLa cells expressing different levels of PIR were pre‐labelled with GFP and co‐cultured with activated CD8^+^ T for 20 hours, followed by flow cytometry analysis. Detached cells were first gated based on forward scatter and side scatter, and further gated by GFP positivity to determine the survival rate of HeLa cells. Data were analyzed using the NovoExpress Software (ACEA Biosciences, Inc).

### Generation of CD8+ T Cells and Cytotoxicity Assay In Vitro

To generate activated effector CD8^+^ T lymphocytes (also known as cytotoxic T lymphocyte, CTL; T‐killer cell), naïve CD8^+^ T cells were isolated from human PBMC with a MojoSort Human CD8 Naïve T Cell Isolation Kit (Biolegend, Cat # 480 045), followed by co‐culture with 60 U mL^−1^ human recombinant IL‐2, 2 µg mL^−1^ anti‐human CD3 antibody and 4 µg mL^−1^ anti‐human CD28 antibody in RPMI‐1640 medium for 3 days. These in vitro activated CD8^+^ CTL were then harvested and used for cytotoxicity assay. GPF^+^ HeLa cells expressing different levels of PIR (2 × 10^5^) were co‐cultured with CD8^+^ CTLs (6 × 10^5^) in RPMI 1640 medium for 20 hours. The morphologies of HeLa cells were imaged by microscopy and the survival rates were determined by flow cytometry.

### Gel Shift Assay (EMSA)

The Gel shift assay was performed according to the method published previously.^[^ ^36^ ^]^ Briefly, an *Ecoli*. purified protein or a mixture of proteins were incubated with a 3′ end‐labelled FAM DNA probe containing the putative protein binding site. And then, the reaction products were analyzed on a no denaturing polyacrylamide gel. The sequence of probe for *FAS* promoter (3′‐2a) was 5′ ‐AACGTCTGTGAGCCTCTCATGTTGCAGCCA‐3′.

### Luciferase Reporter Assay

The pGL3‐based Luciferase reporter plasmids contains a variety of *FAS* promoter truncations as indicated in Figure S3 (Supporting Information) were constructed. HEK293T cells were co‐transfected with these reporters and Flag‐PIR. 24 hours post‐transfection, cells were harvested and measured for luciferase activity using luciferase reporter gene assay kit (Yeasen, China).

### Statistical Analysis

All results reported in this manuscript were presented as means±SD or means±SEM of at least three independent experiments (except for RNA‐seq, n = 2). *p* values were calculated based on unpaired Student's *t* test using Graphpad Prism 8 (Graphpad Software). *p* < 0.05 was considered statistically significant. *, **, *** and n.s. represent *p* < 0.05, *p* < 0.01, *p* < 0.001 and no significant difference, individually, unless otherwise indicated.

### Study Approval

All animal procedures were approved by Animal Care and Use Committee in Xiamen University (Ethics license, XMULAC20180041).

## Conflict of Interest

The authors declare no conflict of interest.

## Author Contributions

H.M., M.S., F.Z. contributed equally to this work. H.M., M.S., B.L. and Q.L. conceived the project. H.M., M.S., and Q.L. designed most experiments. H.M., M.S, F.Z., T.C., S.W., D.S., L.C., and Y.W. performed experiments. F.L., J.W., and B.J. helped with data analysis and discussion. Q.L. and H.M. wrote the manuscript. All authors commented on the manuscript.

## Supporting information

Supporting Information

## Data Availability

Raw sequencing data reported in this manuscript have been deposited at the Genome Sequence Archive at National Genomics Data Center (Bejing, China) under the BioProject ID: PRJCA014787.
