# Supplementary material for: Pirin Inhibits FAS‐Mediated Apoptosis to Support Colorectal Cancer Survival
Source: Adv Sci (Weinh). 2023 Dec 26;11(10):2301476. doi: 10.1002/advs.202301476 (PMC10933653; doi:10.1002/advs.202301476)
Supplement: Supplementary file 1 — Supporting Information [file ADVS-11-2301476-s001.pdf]

ADVANCED SCIENCE
Open Access

Supporting Information

for *Adv. Sci.*, DOI 10.1002/advs.202301476

Pirin Inhibits FAS-Mediated Apoptosis to Support Colorectal Cancer Survival

Huanhuan Ma, Muhammad Suleman, Fengqiong Zhang, Tingyan Cao, Shixiong Wen, Dachao Sun, Lili Chen, Bin Jiang, Yue Wang, Furong Lin, Jinyang Wang, Boan Li and Qinxi Li**

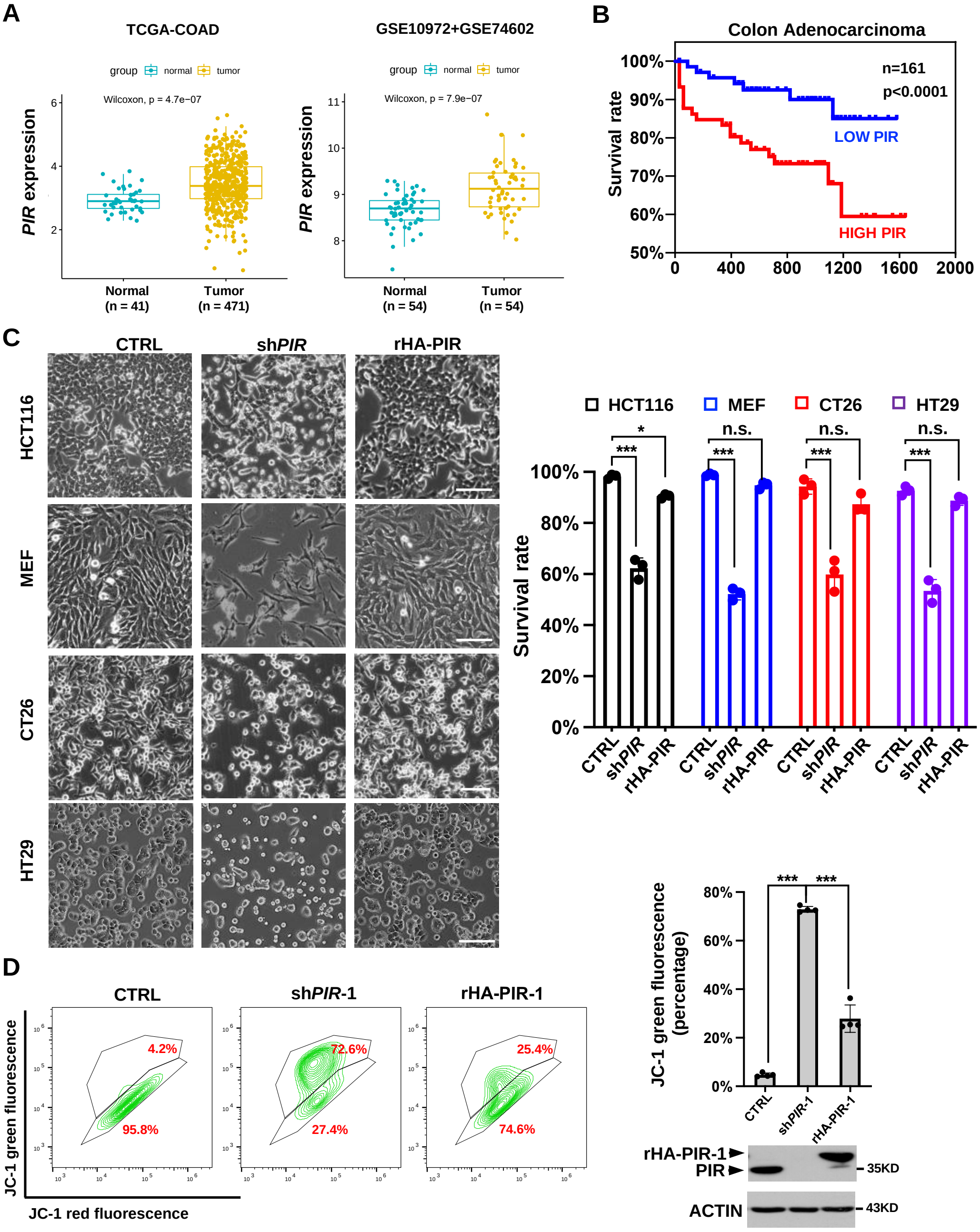

A
TCGA-COAD
group normal tumor
Wilcoxon, p = 4.7e−07
PIR expression
Normal (n = 41)
Tumor (n = 471)
GSE10972+GSE74602
Wilcoxon, p = 7.9e−07
Normal (n = 54)
Tumor (n = 54)
B
Colon Adenocarcinoma
n=161
p<0.0001
Survival rate
LOW PIR
HIGH PIR
C
CTRL
shPIR
rHA-PIR
HCT116
MEF
CT26
HT29
Survival rate
n.s.
CTRL
shPIR
rHA-PIR
D
CTRL
shPIR-1
rHA-PIR-1
4.2%
95.8%
72.6%
27.4%
25.4%
74.6%
JC-1 green fluorescence
JC-1 red fluorescence
JC-1 green fluorescence (percentage)
rHA-PIR-1
PIR
35KD
ACTIN
43KD

Fig. S1. PIR deficiency triggers apoptosis

(**A**) Boxplot showing upregulated PIR expression in colon cancer as compared with normal tissue. *P* value were analyzed by *t*-test. Data were public available in TCGA and GEO database.

(**B**) Kaplan-Meier survival plots of patients with low or high PIR expression in colon adenocarcinoma. Datasets are publicly available from the Oncomine database. Statistical analysis was performed by Log-rank test.

(**C**) HCT116, MEF, CT26 and HT29 cells were pre-expressed for sh*PIR*-resistant PIR (rescuing HA-tagged PIR, rHA-PIR) and further infected with lentivirus-based sh*PIR*. After 72 hours of infection, cell morphology (left panel) and survival rate (right panel) were determined. Scale bars represent 100 µm. Results are presented as mean±SD of three independent experiments (unpaired Student's *t*-test, **p*<0.05, ****p*<0.001, n.s.: no significant difference).

(**D**) HCT116 cells with or without (w/wo) pre-expression of rHA-PIR was knocked down for PIR. After 48 hours of PIR knockdown, cells were incubated with JC-1 for 20 min at 37 ℃ and analyzed for green/total cell ratios with flow cytometry (left panel). Statistical results (right panel) are presented as mean±SD of four independent experiments (unpaired Student's *t*-test, ****p*<0.001).

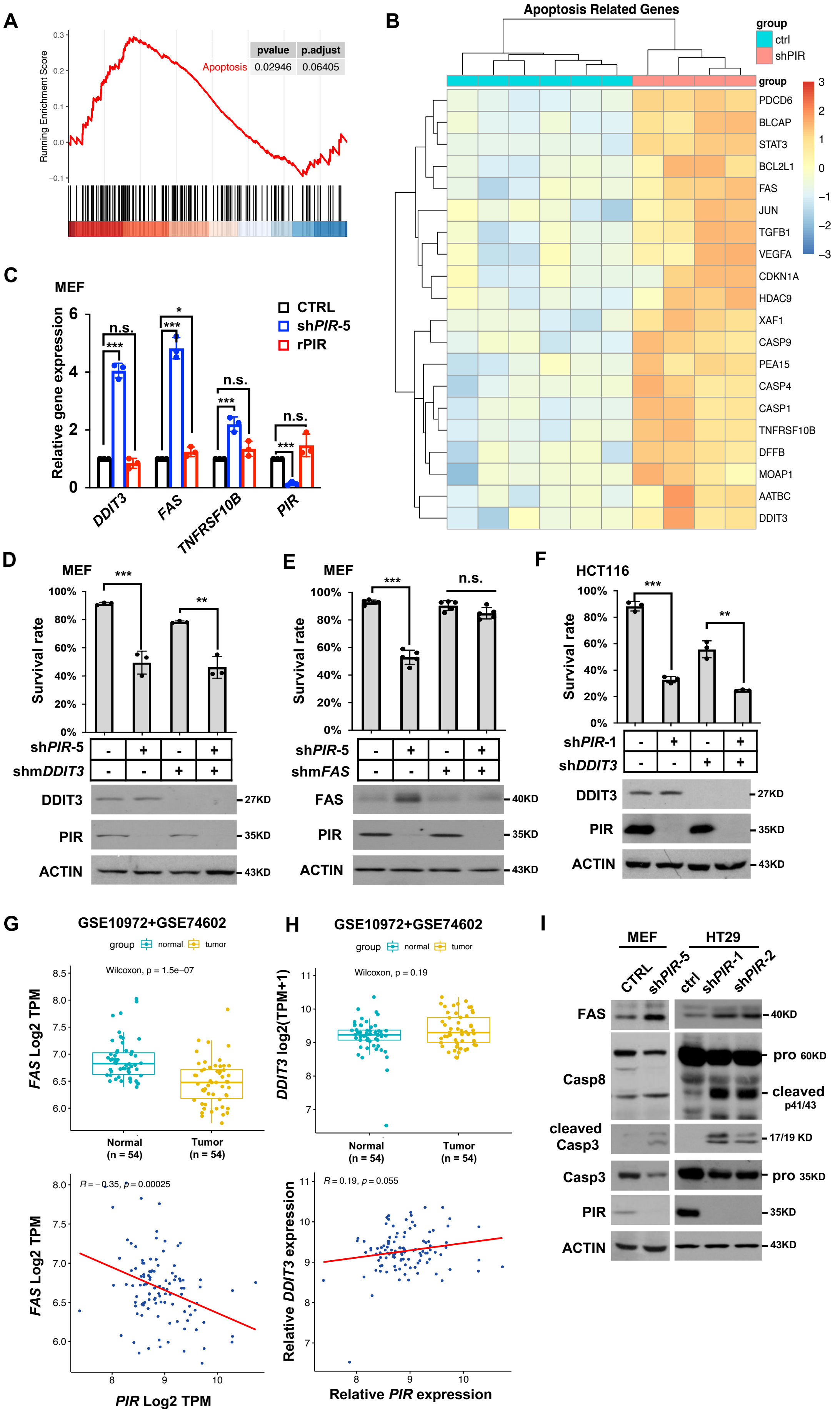
A
Running Enrichment Score
pvalue p.adjust
Apoptosis 0.02946 0.06405
B
Apoptosis Related Genes
group
ctrl
shPIR
PDCD6
BLCAP
STAT3
BCL2L1
FAS
JUN
TGFB1
VEGFA
CDKN1A
HDAC9
XAF1
CASP9
PEA15
CASP4
CASP1
TNFRSF10B
DFFB
MOAP1
AATBC
DDIT3
C
MEF
Relative gene expression
CTRL
shPIR-5
rPIR
DDIT3
FAS
TNFRSF10B
PIR
D
MEF
Survival rate
shPIR-5
shmDDIT3
DDIT3
PIR
ACTIN
27KD
35KD
43KD
E
MEF
Survival rate
shPIR-5
shmFAS
FAS
PIR
ACTIN
40KD
35KD
43KD
F
HCT116
Survival rate
shPIR-1
shDDIT3
DDIT3
PIR
ACTIN
27KD
35KD
43KD
G
GSE10972+GSE74602
group normal tumor
Wilcoxon, p = 1.5e−07
FAS Log2 TPM
Normal (n = 54)
Tumor (n = 54)
R = −0.35, p = 0.00025
FAS Log2 TPM
PIR Log2 TPM
H
GSE10972+GSE74602
group normal tumor
Wilcoxon, p = 0.19
DDIT3 log2(TPM+1)
Normal (n = 54)
Tumor (n = 54)
R = 0.19, p = 0.055
Relative DDIT3 expression
Relative PIR expression
I
MEF
HT29
CTRL
shPIR-5
ctrl
shPIR-1
shPIR-2
FAS
Casp8
cleaved Casp3
Casp3
PIR
ACTIN
40KD
pro 60KD
cleaved p41/43
17/19 KD
pro 35KD
35KD
43KD

Fig. S2. FAS pathway is involved in PIR deficiency-initiated cell death

(**A**) GSEA enrichment of the RNA-Seq data public available PIR KD expression dataset (GSE17551 and GSE16798) for signatures of apoptosis pathway. *P* values were determined by one-tailed permutation test by GSEA.

(B) Expression heatmap of the most upregulated apoptotic genes analyzed using public available PIR KD expression dataset (GSE17551 and GSE16798).

(**C**) MEF cells with or without rHA-PIR expression were knocked down for PIR. 48 hours later, relative mRNA levels of indicated genes were determined by RT-qPCR (left). Data are shown as mean±SEM and analyzed using unpaired Student's *t*-test (n=3, **p*<0.05, ****p*<0.001).

(**D**) MEF cells were knocked down for PIR and DDIT3 alone or in combination. After 72 hours of knockdown, cells were stained with PI for 10 min and then subjected to survival analysis by flow cytometry. Data represent mean±SD of three independent experiments (unpaired Student's *t*-test, ****p*<0.001).

(**E**) MEF cells were knocked down for PIR and FAS alone or in combination. 72 hours later, the survival rates were determined by using flow cytometry after PI staining. Data represent mean±SD of five independent experiments (***p*<0.01, ****p*<0.001).

(**F**) HCT116 cells were knocked down for *PIR* and *DDIT3* alone or in combination. 72 hours later, the survival rates were determined by using flow cytometry after PI staining. Data represent mean±SD of three independent experiments (***p*<0.01, ****p*<0.001).

(**G**) Boxplot (upper) showing FAS expression is downregulated in colon cancer compared to its normal adjacent tissue. Center line indicates the median, lower and upper hinges represent the 25th and 75th percentiles, respectively. *P* value were analyzed by *t*-test. The correlation between FAS and PIR were shown in lower panel. Correlation coefficient and *p* value were analyzed by *pearson*.

(**H**) Boxplot (upper) showing that DDIT3 expression is not changed in colon cancer as compared with corresponding adjacent normal tissue. *P* value were analyzed by Student's *t*-test. The correlation between expression levels of DDIT3 and PIR were shown in lower panel. Correlation coefficient and *p* value were analyzed by *pearson.*

(**I**) PIR knockdown MEF and HT29 cells were detected for expression of indicated proteins to evaluate the upregulation of FAS and cleavage of caspase 3/8.

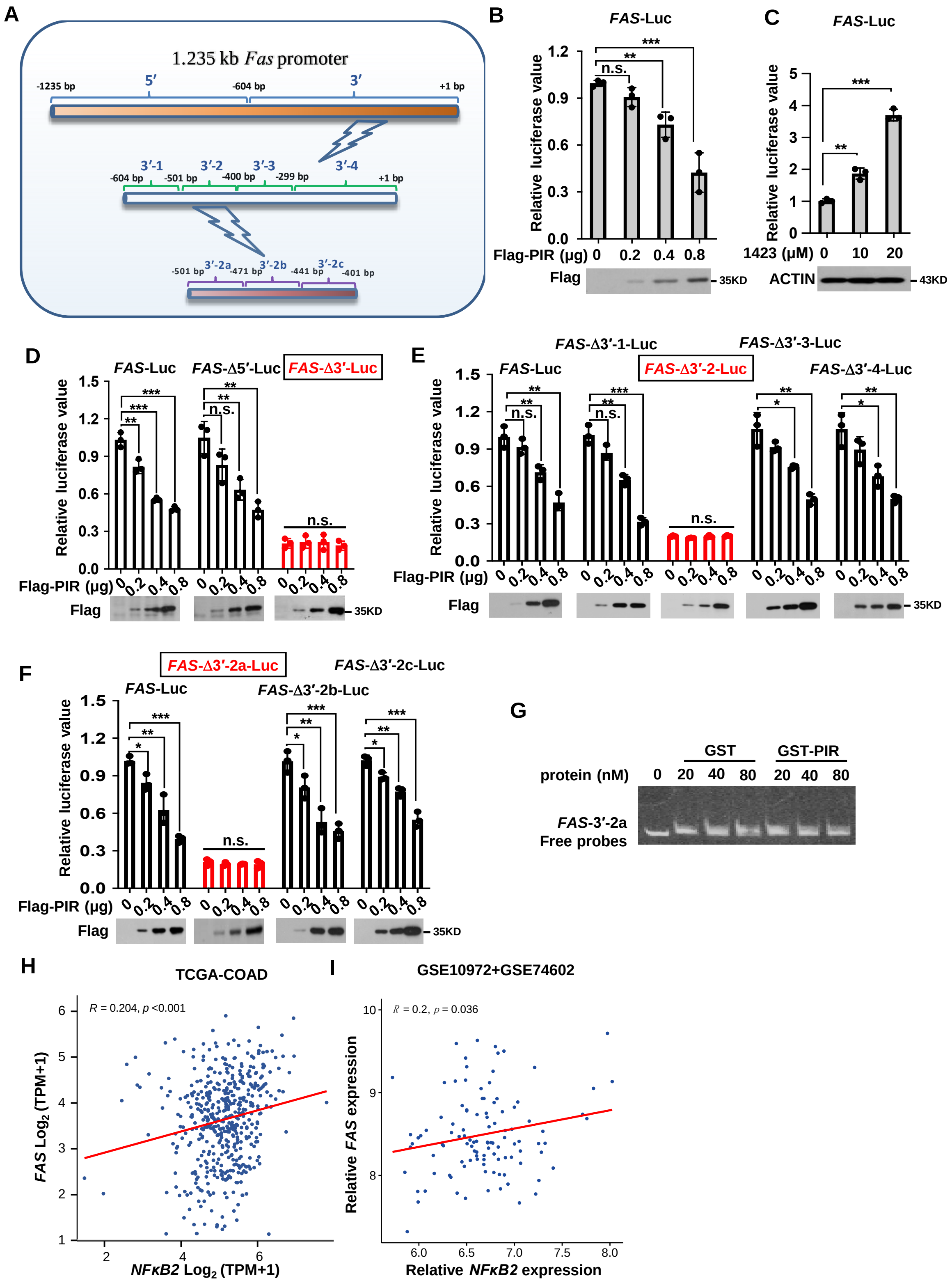
A
1.235 kb Fas promoter
B
FAS-Luc
Relative luciferase value
Flag-PIR (μg)
Flag
35KD
C
FAS-Luc
1423 (μM)
ACTIN
43KD
D
FAS-Luc
FAS-Δ5′-Luc
FAS-Δ3′-Luc
E
FAS-Δ3′-1-Luc
FAS-Δ3′-2-Luc
FAS-Δ3′-3-Luc
FAS-Δ3′-4-Luc
F
FAS-Δ3′-2a-Luc
FAS-Δ3′-2b-Luc
FAS-Δ3′-2c-Luc
G
protein (nM)
GST
GST-PIR
FAS-3′-2a
Free probes
H
TCGA-COAD
R = 0.204, p <0.001
FAS Log2 (TPM+1)
NFκB2 Log2 (TPM+1)
I
GSE10972+GSE74602
R = 0.2, p = 0.036
Relative FAS expression
Relative NFκB2 expression

Fig. S3. NFκB2 is required for *FAS* transactivation

(**A**) A schematic diagram depicting information of full-length *FAS* promoter and its various deletion mutants.

(**B**) HEK293T cells were co-transfected with *FAS*-Luc and increasing doses of Flag-PIR. After 24 hours of transfection, luciferase activities were determined and normalized to the control (first column). Data are presented as mean±SD of three independent experiments. (n=3, unpaired Student's *t*-test, **p<0.01, ***p<0.001, n.s.: no significance).

(**C**) HEK293T cells were transfected with *FAS*-Luc and treated with PIR inhibitor CCG-1423 for 24 hours. Luciferase activities were determined and analyzed as in (B).

(**D** to **F**) HEK293T cells were co-transfected with increasing doses of Flag-PIR and each of luciferase reporter vectors containing various deletions of *FAS* promoter as indicated 24 hours later, luciferase activities were determined and normalized to the first column accordingly. Data represent mean±SD.(n=3, unpaired Student's *t*-test, *p<0.05, **p<0.01, ***p<0.001, n.s. no significance).

(**G**) Gel shift assay was performed with *FAS*-3'-2a probe and increasing concentrationsof GST-PIR protein. GST protein was used as a negative control. *FAS*-3'-2a probe wasdiluted to a final concentration of 20 nM.

(**H**) The correlation analysis of mRNA level between NF-κB2 and FAS in colon adenocarcinoma data set (TCGA-COAD).

(**I**) The correlation analysis of mRNA level between *DDIT3* and *PIR* in GEO data set (GSE10972 and GSE74602).

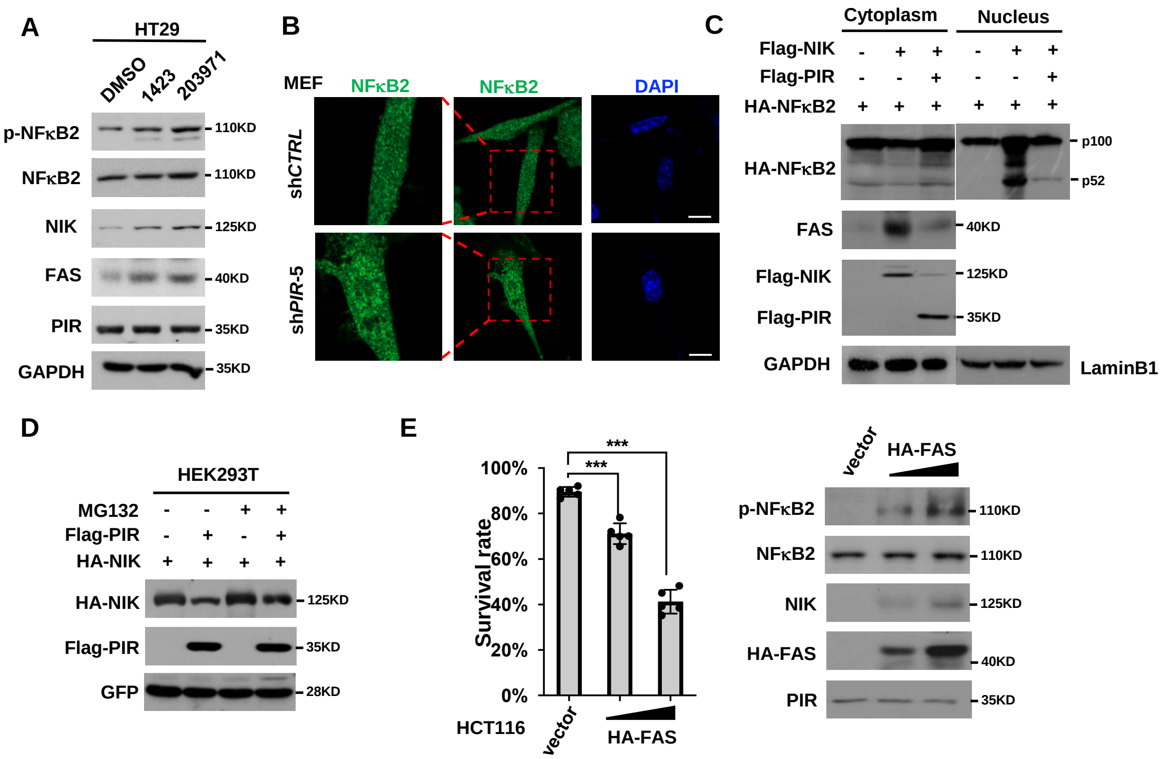
A
HT29
DMSO
1423
203971
p-NFκB2
NFκB2
NIK
FAS
PIR
GAPDH
110KD
110KD
125KD
40KD
35KD
35KD
B
MEF
NFκB2
NFκB2
DAPI
shCTRL
shPIR-5
C
Cytoplasm
Nucleus
Flag-NIK
Flag-PIR
HA-NFκB2
HA-NFκB2
p100
p52
FAS
40KD
Flag-NIK
125KD
Flag-PIR
35KD
GAPDH
LaminB1
D
HEK293T
MG132
Flag-PIR
HA-NIK
HA-NIK
125KD
Flag-PIR
35KD
GFP
28KD
E
***
***
100%
80%
60%
40%
20%
0%
Survival rate
HCT116
vector
HA-FAS
vector
HA-FAS
p-NFκB2
110KD
NFκB2
110KD
NIK
125KD
HA-FAS
40KD
PIR
35KD

Fig. S4. PIR inhibits FAS membrane translocation and NFκB2 activation

(**A**) HT29 cells were separately treated with 5 μM of PIR inhibitors CCG-1423 and CCG-203971 for 12 hours and then detected for indicated proteins.

(**B**) Immunostaining was performed to determine the nuclear localization of NFκB2 (green) in MEF cells with or without PIR knockdown. Scale bars represent 20 μm.

(**C**) HEK293T cells were transfected with indicated plasmids alone or in combinations. 24 hours later, cells were fractionated to obtain nuclear and cytoplasmic fractions, followed by detection of indicated proteins.

(**D**) 293T cells were transfected with indicated plasmids. 24 hours post-transfection, cells were treated with or without 10 μM MG132 for 8 hours and then detected for the expression of indicated protein.

(**E**) HCT116 cells were transfected with increasing doses of HA-FAS. 72 hours post-transfection, cells were subjected to flow cytometry for survival rate analysis (left panel) and WB (right panel) for indicated protein detection.

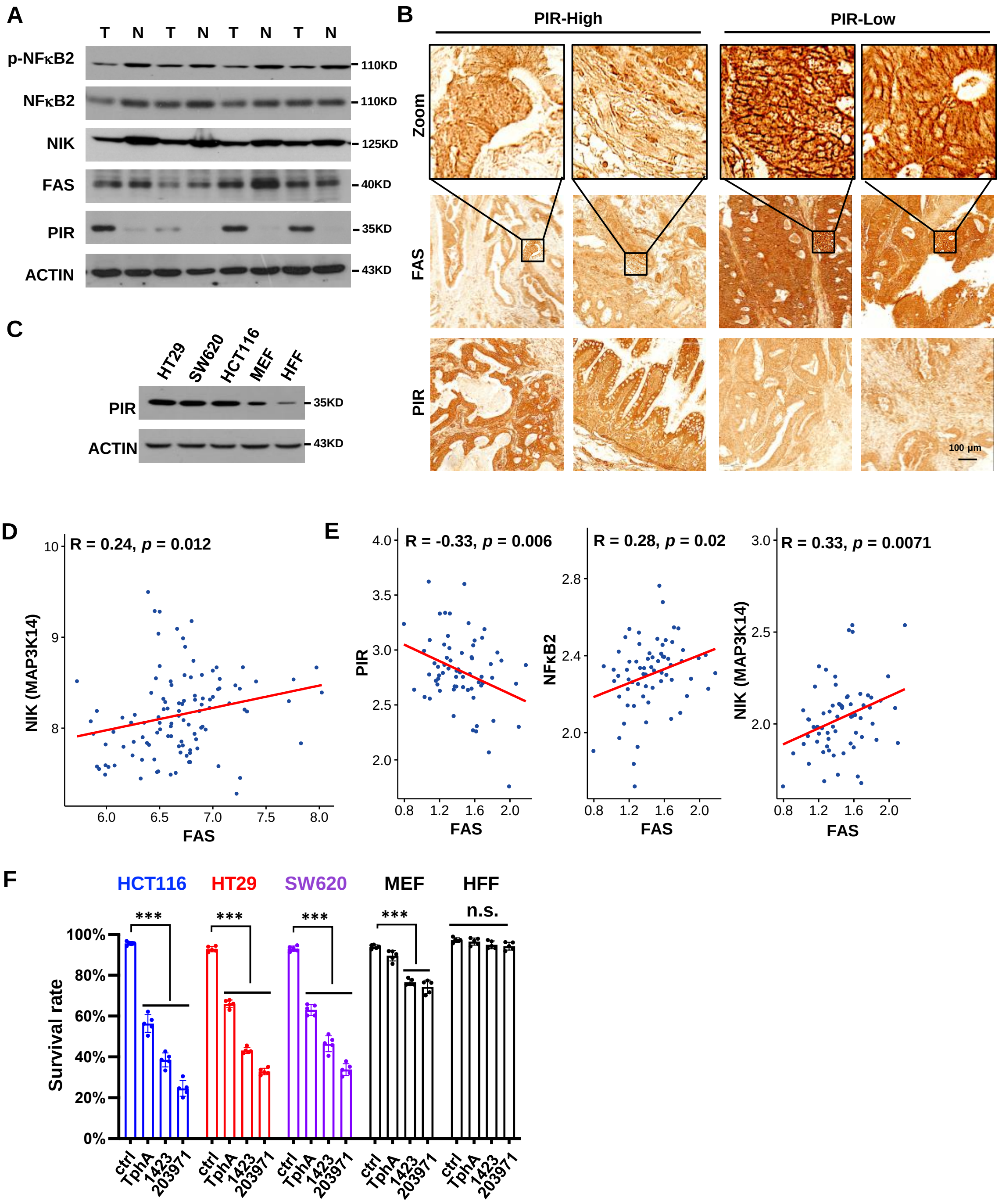

A
T N T N T N T N
p-NFκB2
NFκB2
NIK
FAS
PIR
ACTIN
110KD
110KD
125KD
40KD
35KD
43KD
B
PIR-High
PIR-Low
Zoom
FAS
PIR
100 μm
C
HT29
SW620
HCT116
MEF
HFF
PIR
ACTIN
35KD
43KD
D
R = 0.24, p = 0.012
NIK (MAP3K14)
FAS
E
R = -0.33, p = 0.006
R = 0.28, p = 0.02
R = 0.33, p = 0.0071
PIR
NFκB2
NIK (MAP3K14)
FAS
F
HCT116
HT29
SW620
MEF
HFF
***
n.s.
Survival rate
ctrl
TphA
1423
203971

Fig. S5. PIR facilitates colon cancer formation by inhibiting FAS expression

(**A**) CRC samples were subjected to WB for indicated proteins. T: Hcol-Ade carcinoma, N: tumor-adjacent normal colon tissue.

(**B**) Human colon cancer samples were analyzed for PIR and FAS expression by immunochemistry (IHC). Scale bar represents 100 μm.

(**C**) WB was performed to determine the expression of PIR in various cell.

(**D**) Correlation coefficient and *p* value between FAS and MAP3K14 (NIK) were analyzed by *pearson*.

(**E**) Correlation coefficient and p value of indicated genes were analyzed by *pearson*.

(**F**) Survival analysis of indicated cell lines treated with or without PIR inhibitors (TphA, CCG-1423 and CCG-203971) for 48 hours. Data are shown as mean±SD.(n=5, unpaired Student's *t*-test, $^{**}p<0.01$, $^{***}p<0.001$)

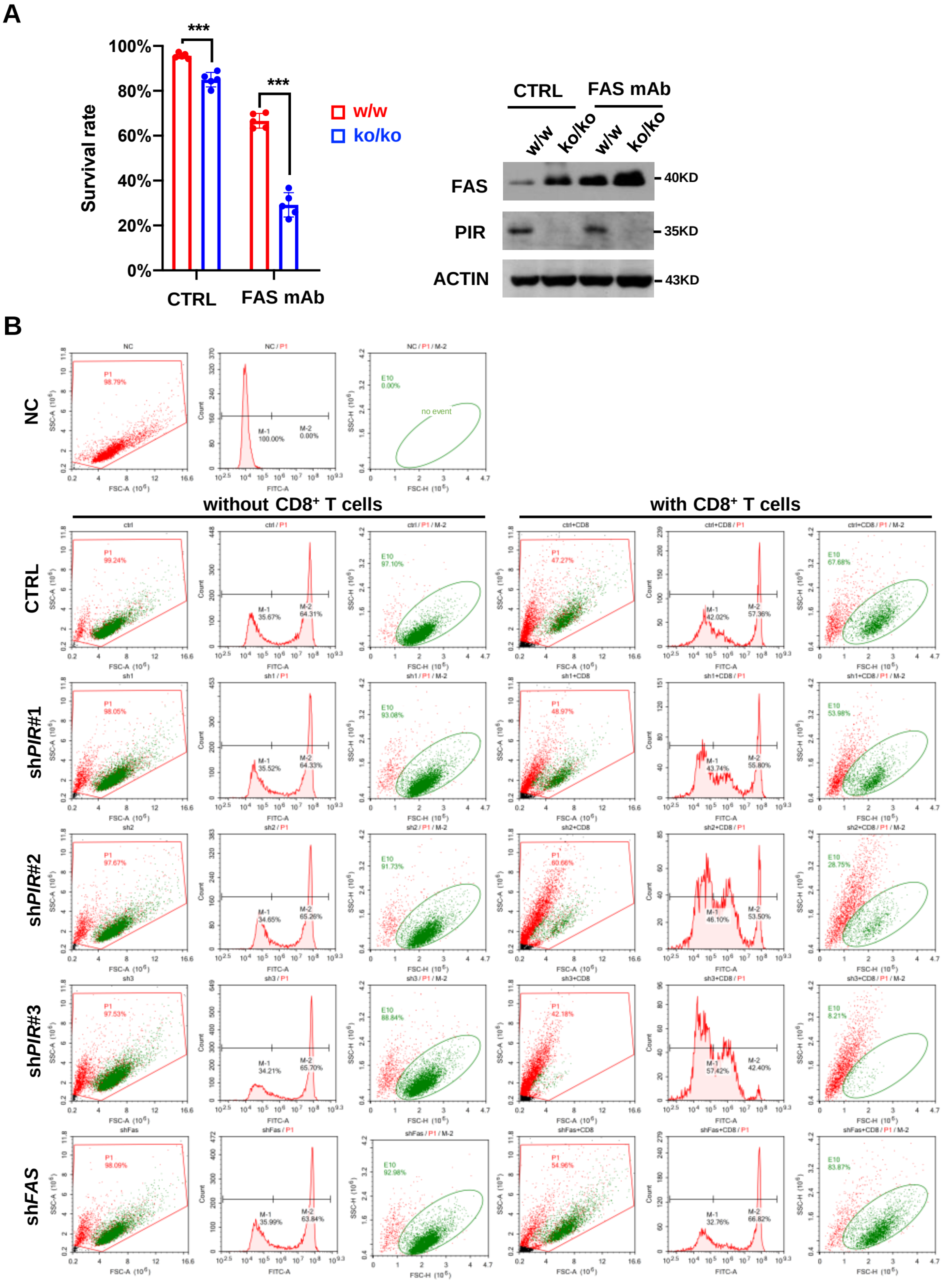

Fig. S6. PIR inhibition sensitizes cells to FAS-based immunotherapy

(**A**) PIR KO MEFs were created by expressing adenovirus-based Cre recombinase in primary MEFs derived from PIR cKO mice and treated w/wo anti-FAS-mAb (10 μg mL^{-1}) for 24 hours, followed by detection of survival rate (n=5, unpaired Student's *t*-test, ****p*<0.001).

(**B**) Representative flow cytometry data of Fig. 6C. P1 is gated for excluding the debris. M-2 is gated for GFP-labeled HCT116 cells (T cells and HCT116 cells with low-GFP expression were excluded).

A

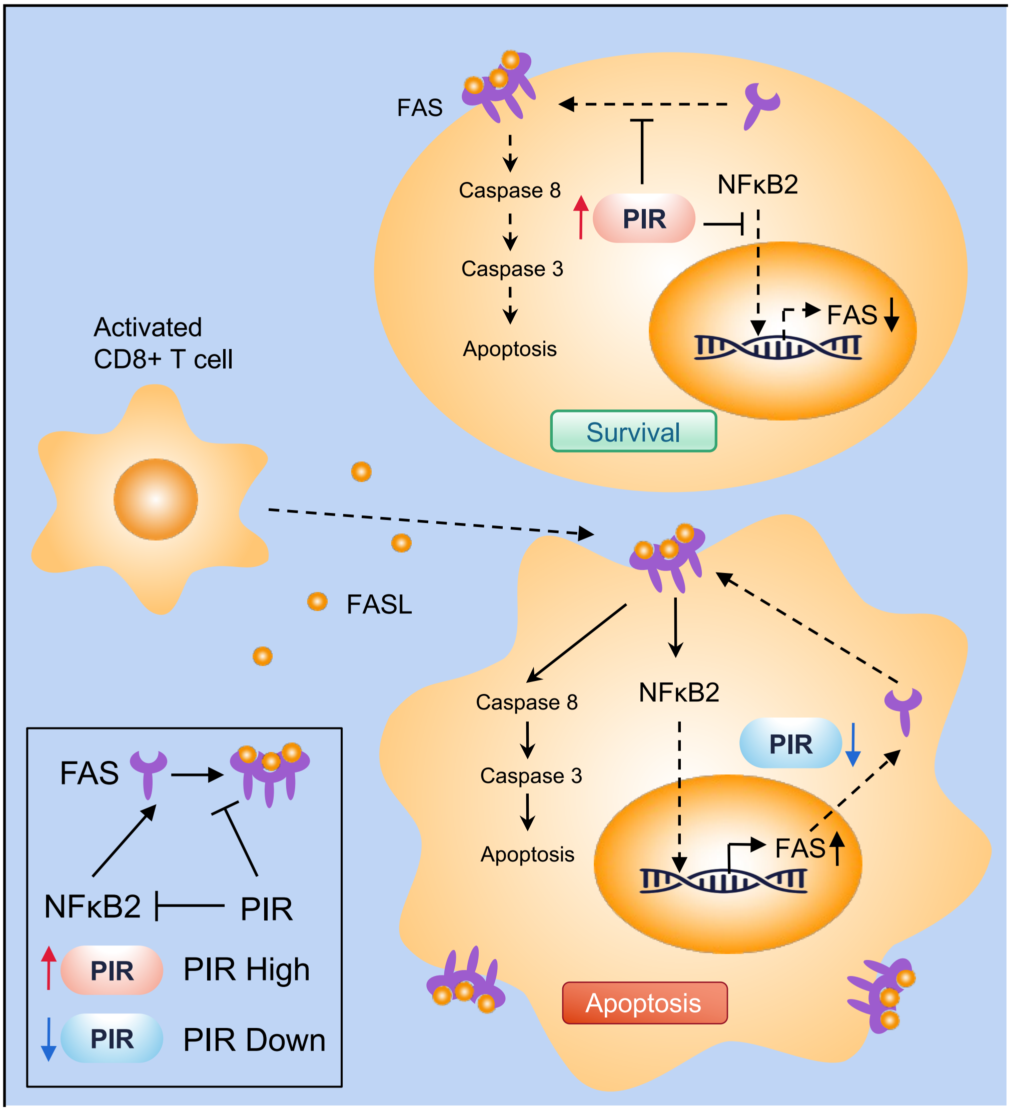

Fig. S7. PIR suppresses FAS-dependent apoptosis by switching off NFκB2-FAS axis

(**A**) A working model describing PIR suppresses FAS-dependent cell death by switching off NFκB2-FAS axis.
